# Is It Cost‐Effective to Induce Labour Early to Prevent Shoulder Dystocia? Evidence From the Big Baby Trial

**DOI:** 10.1111/1471-0528.18160

**Published:** 2025-05-01

**Authors:** Seyran Naghdi, Stavros Petrou, Martin Underwood, Sanjeev Deshpande, Siobhan Quenby, Lauren Ewington, Jason Gardosi, Hema Mistry

**Affiliations:** ^1^ Warwick Clinical Trials Unit University of Warwick Coventry UK; ^2^ Nuffield Department of Primary Care Health Sciences University of Oxford Oxford UK; ^3^ University Hospitals Coventry and Warwickshire Coventry UK; ^4^ Shrewsbury and Telford Hospitals NHS Trust Telford UK; ^5^ Warwick Biomedical Sciences University of Warwick Coventry UK; ^6^ Warwick Medical School University of Warwick Coventry UK; ^7^ Perinatal Institute Birmingham UK

**Keywords:** economics of health care, labour: induction, obstetrics and gynaecology

## Abstract

**Background:**

The cost‐effectiveness of early induction of labour for suspected large‐for‐gestational‐age foetuses to prevent shoulder dystocia is unknown.

**Methods:**

A within‐trial economic evaluation of induction at 38 + 0 to 38 + 4 weeks' gestation for suspected large‐for‐gestational‐age foetuses. Resource use and costs were measured to 6 months postpartum. We estimated incremental cost per case of shoulder dystocia prevented and incremental cost per maternal quality‐adjusted life year (QALY) gained. We collected data for planned caesarean sections in a cohort study.

**Findings:**

Mean combined woman and infant costs in the induction arm were £89 (95% confidence interval (CI): −£79, £257) higher than the standard care arm, driven by increased neonatal costs. The incremental cost of preventing one case of shoulder dystocia was £11 879 and the incremental cost per maternal QALY gained was £39 518. The probability of early induction being cost‐effective was 0.65 at a cost‐effectiveness threshold of £20 000 per case of shoulder dystocia prevented, but 0.36 at a cost‐effectiveness threshold of £20 000 per maternal QALY gained. The cohort study found the mean cost was £310 (95% CI: £74, £545) higher in the induction arm than in the planned caesarean group.

**Interpretation:**

Early induction of labour increased neonatal care costs. It is not a cost‐effective approach when effects are restricted to maternal QALYs. Planned caesarean section might be cost‐saving when compared to early induction, although we did not assess longer‐term effects such as an increased risk of repeat caesarean sections. Assessments of long‐term effects on the mother and infant should be incorporated into future studies.

**Trial Registration:** ISRCTN18229892

## 1 Introduction

Shoulder dystocia is defined as a vaginal cephalic delivery that requires additional obstetric manoeuvres to deliver the fetus after the head has delivered and gentle traction has failed. This may lead to complications such as maternal haemorrhage and third/fourth‐degree perineal tears [1]. Complications for the neonate include fracture of the clavicle or humerus, brachial plexus injury, hypoxic–ischemic encephalopathy, or neonatal death [2]. A 2023 Healthcare Safety Investigation Branch report found that 11% of babies with hypoxic–ischemic encephalopathy were delivered after shoulder dystocia [3]. Macrosomia (birth weight > 4 kg) and large‐for‐gestational age (LGA) (> 90th centile) babies are at increased risk of shoulder dystocia [4].

Early induction of labour may reduce neonatal weight and therefore mitigate the risk of shoulder dystocia but can increase the risk of other complications. The effectiveness of early induction on reducing shoulder dystocia, and the effects on maternal and neonatal morbidity, are uncertain. The 2012 Royal College of Obstetricians and Gynaecologists Green‐Top guideline found limited support for early delivery of LGA foetuses to reduce shoulder dystocia. A subsequent 2016 systematic review (*n* = 1190 from four studies) found that induction reduced the incidence of shoulder dystocia by 40% (relative risk (RR): 0.60, 95% confidence interval (CI) 0.37 to 98.0) and a 2017 review including the same four trials found a similar effect size but concluded that this was not statistically significant [5, 6]. The Big Baby trial randomised 2893 women with an LGA fetus to early induction of labour or standard care [7]. The analysis found a statistically non‐significant reduction of shoulder dystocia from 3.1% to 2.3% (RR: 0.75, 95% CI: 0.48–1.17) by ‘intention‐to‐treat’ but a significant reduction from 3.7% to 2.3% (RR: 0.62, 95% CI: 0.41–0.92) in the pre‐specified ‘per‐protocol’ analysis, which excluded the large proportion of early deliveries in the control arm. Women in the induction arm had fewer operative deliveries and postpartum haemorrhages but longer hospital stays [7]. There were no statistically significant differences in secondary neonatal outcomes [7].

Very few studies have evaluated the cost‐effectiveness of strategies to prevent shoulder dystocia and brachial plexus injuries [8, 9]. An economic decision analysis in the USA found that expectant management of a 4.5 kg infant, which cost $4014 per injury‐free child, compared to $5212 for elective caesarean birth and $5165 for induction of labour at 38–39 weeks (price date unspecified) [10]. Additionally, complications arising from shoulder dystocia are common reasons for litigation. Between 2000 and 2010 in England, settlements for 250 cases exceeded £100 million, translating to around £400 000 per case [11]. As then, costs have increased so that the current compensation cost to the National Health Service (NHS) for an infant who has long‐term severe brain injury is on average £13.5 million [12]. However, the actual costs, incorporating the impact on infants, families, and the NHS, are immeasurable [12]. To our knowledge, the cost‐effectiveness of early induction of labour in comparison to standard care to prevent shoulder dystocia for suspected LGA foetuses has not been studied in a randomised controlled trial (RCT) in the UK, and therefore our study aimed to undertake this assessment.

## 2 Methods

### 2.1 Trial Background

The Big Baby trial was a prospective, multi‐centre RCT. The trial randomised women between June 2018 and November 2022, from 106 participating UK hospitals [7, 13]. Details of the trial are presented elsewhere [7, 13]. In brief, women with a suspected LGA fetus (estimated fetal weight (EFW) > 90th customised centile), between 35 + 0‐ and 38 + 0‐weeks, were eligible for inclusion. Consenting women were randomly assigned 1:1 to either planned induction of labour between 38 + 0 and 38 + 4 weeks or to standard care via an online web application accessible to all recruiting sites. Women were randomised using minimisation, balancing site, estimated fetal weight centile (EFW) (≤ 95th, > 95th) and maternal age (≤ 35, > 35 years of age) [7]. The primary clinical outcome was the incidence of shoulder dystocia, assessed by an independent expert panel reviewing birth records. Secondary outcomes included fetal and maternal peripartum outcomes, patient‐reported outcomes at 2 and 6 months postpartum, and women's health‐related quality of life (HRQoL) [13]. Eligible women who declined the trial and opted for planned caesarean birth after reading the patient information sheet were also followed up.

### 2.2 Overview of Economic Evaluation

The economic evaluation aimed to assess the cost‐effectiveness of early induction of labour (at 38 + 0 to 38 + 4 weeks' gestation) compared with standard care in women with LGA foetuses. An NHS and personal social services (PSS) perspective was adopted, aligning with recommendations from the National Institute for Health and Care Excellence [14]. The primary analysis included a cost‐effectiveness assessment, expressed as the incremental cost per case of shoulder dystocia prevented, and a cost‐utility analysis, expressed as the incremental cost per maternal quality‐adjusted life year (QALY) gained. The time horizon extended from randomisation to 6 months postpartum. No discounting of costs and outcomes was undertaken due to the short time horizon.

### 2.3 Measurement and Valuation of Resource Use

Health service resource use data collected from randomisation to hospital discharge post‐birth included unscheduled hospital visits, induction of labour, mode of delivery, medications, equipment usage, labour complications and adverse events for women. For infants, recorded resources included hospital care type and duration, hospital transfers, neonatal procedures, tests and medications.

Follow‐up questionnaires at 2 and 6 months postpartum provided additional resource use data, including hospital readmissions, outpatient visits, accident and emergency attendances, medication usage and community health and social service utilisation. Collected data were cross‐referenced with case record forms to avoid duplication based on date and reason, where applicable.

Unit costs from key databases were used to value resource inputs. Costs for women, including antenatal, intrapartum, and postnatal care, were determined using the maternity pathway from the National Tariff Workbook (April 2022) [15]. For infants, hospital admission and outpatient visit costs were based on the National Schedule of NHS costs for the year 2021/22 [16]. Community‐based health and social care service costs were valued using unit cost data from the Personal Social Services Research Unit (PSSRU) Unit Costs of Health and Social Care 2022 compendium [17]. Hospital‐based doctor appointments were based on a 15‐min duration, while appointments with other healthcare staff were estimated to be 30 min. For other healthcare staff, hourly costs were estimated using their average salary, employer on‐costs and overheads. Medication costs were obtained from the British National Formulary and the Prescription Cost Analysis database, with costs computed based on dosages and frequencies [18, 19]. All monetary values were reported in pounds sterling for the price year 2021–22 [15]. Unit cost details are presented in the Appendix S2: Table S1.

### 2.4 Measurement of Outcomes

For infants, the primary outcome for the economic evaluation focused on the incremental cost of preventing one case of shoulder dystocia.

The primary health outcome for women was maternal QALYs, a measure combining survival and HRQoL [20]. HRQoL was evaluated at baseline and at 2 and 6 months postpartum using the EuroQol‐5 Dimensions five‐level (EQ‐5D‐5L) instrument, which covers mobility, self‐care, usual activities, pain/discomfort, and anxiety/depression, each with five response levels [21]. EQ‐5D‐5L responses were converted into utility values using the Hernandez‐Alava et al. algorithm [22]. QALYs were computed by calculating the area under the baseline‐adjusted utility curve, assuming linear interpolation between health utility measurements at different time points [22]. Health utility and QALY estimates over the trial were summarised by arm and timepoint, presented as means with standard errors. Differences by arm were analysed using a two‐sample *t*‐test.

### 2.5 Handling of Missing Data

We employed a fully conditional multiple imputation by chained equations (MICE) package in Stata 18.0 [23] to handle missing cost and health utility data at each time point. Assuming missing data occurred at random (MAR), we assessed this assumption by examining missing data patterns and comparing participant characteristics with complete and missing data [24]. Regression models with covariates including maternal age, fetal weight centile, body mass index (BMI), and recruitment site were used to create 50 multiple imputed datasets given that we had an average of 48% missing data. These predicted missing values for costs and EQ‐5D‐5L utility scores. Bivariate regressions using seemingly unrelated regression (SUREG) models were independently conducted on each imputed dataset to estimate costs, cases of shoulder dystocia, and maternal QALYs for each treatment arm.

### 2.6 Cost‐Effectiveness and Cost‐Utility Analyses

Continuous outcomes including costs and maternal QALYs were analysed using SUREG to estimate adjusted mean differences, controlling for maternal age, fetal weight centile and recruitment site, and reported with 95% CIs. For shoulder dystocia, modified Poisson regression with robust standard errors was used to estimate relative risks (RRs) with 95% CIs.

Cost‐effectiveness results are presented as incremental cost‐effectiveness ratios (ICERs), where the mean additional cost is divided by the mean additional benefit and expressed as (1) incremental cost per case of shoulder dystocia prevented and (2) incremental cost per maternal QALY gained. Standard care served as the reference.

The primary economic evaluation used an intention‐to‐treat (ITT) framework with imputed data. Non‐parametric bootstrapping was employed to generate joint distributions of costs, health outcomes (cases of shoulder dystocia, maternal QALYs), and differences in costs and health outcomes across 3000 bootstrap samples. For each sample, mean values and 95% CIs were computed for incremental costs, prevented cases of shoulder dystocia, and maternal QALYs gained. Rubin's rules were applied to combine estimates across imputed datasets, providing overall mean estimates with standard errors (SE) for costs, prevented shoulder dystocia cases, and maternal QALYs, considering variability within and across imputations [25]. Model validation included assessing the distributions of imputed and observed values.

Visual representation of cost‐effectiveness outcomes involved scatterplots using non‐parametric bootstrapped replications. Cost‐effectiveness acceptability curves (CEACs) illustrated the probability of early induction being cost‐effective compared to standard care at various cost‐effectiveness thresholds. Net monetary benefit (NMB) was estimated across specific cost‐effectiveness thresholds (£20 000 and £30 000 per maternal QALY), with a positive NMB indicating that early induction was cost‐effective. For the primary clinical outcome of shoulder dystocia cases prevented, we found no published cost‐effectiveness threshold values, and we therefore applied hypothetical benchmarks (£5000, £10 000, £20 000 per case). All analyses were conducted using Stata version 18.0 [23].

### 2.7 Sensitivity, Per‐Protocol and Subgroup Analyses

Pre‐specified sensitivity analyses were undertaken to assess the impacts of uncertainty surrounding components of the economic evaluation. This included restricting the analyses to complete cases (i.e., the sample of participants with no missing costs or outcome data at any time point), replicating the cost‐utility analysis using the van Hout crosswalk algorithm for utility estimation instead of the algorithm by Hernandez‐Alava et al. [22, 26], and using a modified ITT (excluding eight participants with a missing primary outcome). A post hoc ‘per‐protocol’ economic analysis (Clinical Estimand 2) was also conducted excluding participants in the standard care arm who received the intervention and those in the intervention arm who did not receive it. This aimed to provide insights into the interpretation of the statistically significant reduction in the incidence of shoulder dystocia observed in this pre‐specified clinical comparison. Subgroup analyses were performed by categorising participants according to their BMI (< 25, ≥ 25), and to their EFW centile (≤ 95th centile, > 95th centile).

### 2.8 Additional Analysis

Data for those eligible women who declined randomisation were collected in a parallel cohort study, some of whom opted for a planned caesarean section. Hence, we also calculated the incremental cost of a planned caesarean section compared to early induction and standard care.

## 3 Results

### 3.1 Study Population and Data Completeness

The Big Baby trial randomised 2893 women to either early induction (*n* = 1447) or standard care (*n* = 1446). Details of the study population and clinical results are reported elsewhere [7]. Appendix S2: Table S2 summarises missing health economics data for both arms at 2 and 6 months postpartum. The small number of participants with complete data for the entire follow‐up duration is due to our approach to categorising ‘missing’ data. For instance, if a participant reported contacts with a health visitor at 2 months postpartum but did not specify the number of contacts, we considered their data incomplete, despite recording all other resource use items fully.

### 3.2 Healthcare Resource Use

Appendix S2: Table S3 presents resource use values for complete cases categorised by treatment arm and follow‐up period. The values include subcategories such as hospital care, community care, and prescribed medications for women and infants. At 2 months postpartum, significant differences were noted in the mean number of community nurse visits for women (mean difference (MD) = 0.037, 95% CI: 0.004 to 0.071, *p*‐value = 0.038). The early induction arm showed significantly fewer walk‐in centre visits for infants compared to standard care (MD = 0.042, 95% CI: −0.082 to −0.001, *p*‐value = 0.038). Infants who were in the standard care arm had significantly more contacts with a midwife than those in the early induction arm (MD = 0.208, 95% CI: 0.031 to 0.385, *p*‐value = 0.022). No significant differences were observed between the arms for other resource use items at 2 or 6 months postpartum.

### 3.3 Economic Costs

Antenatal costs, spanning from randomisation to the admission for birth, were significantly lower in the induction arm compared to the standard care arm (MD = £39.82, 95% CI: −£55.70 to −£23.93, *p* < 0.01). Delivery costs were higher in the induction arm but not statistically significant (MD = £52.01, 95% CI: −£97.72 to £201.75, *p* = 0.47). Postnatal maternal costs at 2 and 6 months postpartum were lower in the early induction arm compared to standard care (MD = £9.99, 95% CI: −£83.33 to £63.35, *p* = 0.79; MD = £6.90, 95% CI: −£34.60 to £20.80, *p* = 0.62, respectively). Total infant costs were significantly higher for the early induction arm (MD = £330.98, 95% CI: £42.44 to £619.52, *p* = 0.02). The adjusted total combined cost difference (£326.28) was not statistically significant (95% CI: −£23.07 to £675.63, *p* = 0.06) (Appendix S1: Table S1). In the per‐protocol analysis, the combined mean cost difference was £291 (95% CI: −£112, £695), driven by increased neonatal costs [£262.41 (95% CI: £76, £601)]. Additional cost details are provided in Appendix S2: Table S4.

### 3.4 Health Related Quality of Life Outcomes

Table 1 summarises EQ‐5D utility scores for each treatment arm at different time points. Women in the early induction arm had a slight non‐significant increase in QALYs (MD = 0.001, 95% CI: −0.005 to 0.008, *p*‐value = 0.69).

**TABLE 1:** EQ‐5D‐5L utility results (ITT population, complete case).

Quality of life	Treatment arm				Between‐arm differences (95% CI)			
	Mean (SE) EQ‐5D index score							
	*N* ^a^	Early induction (*n* = 1447)	*N* ^a^	Standard care (*n* = 1446)	Unadjusted	*p*	Adjusted^b^	*p*
EQ‐5D‐5L utility								
Baseline	1433	0.766 (0.005)	1417	0.761 (0.005)	0.005 (−0.010 to 0.020)	0.52	0.005 (−0.010 to 0.020)	0.51
Two months	957	0.883 (0.004)	812	0.879 (0.004)	0.003 (−0.009 to 0.16)	0.61	0.003 (−0.009 to 0.16)	0.61
Six months	774	0.880 (0.005)	620	0.875 (0.005)	0.005 (−0.010 to 0.020)	0.53	0.005 (−0.010 to 0.020)	0.51
QALYs	696	0.431 (0.002)	534	0.430 (0.002)	0.001 (−0.005 to 0.008)	0.71	0.001 (−0.005 to 0.008)	0.69

^a^ Number of participants with complete data.

^b^ Adjusted for maternal age, fetal weight centile, and recruitment site.

### 3.5 Cost‐Effectiveness Results: Base‐Case Analysis

**FIGURE 1:**
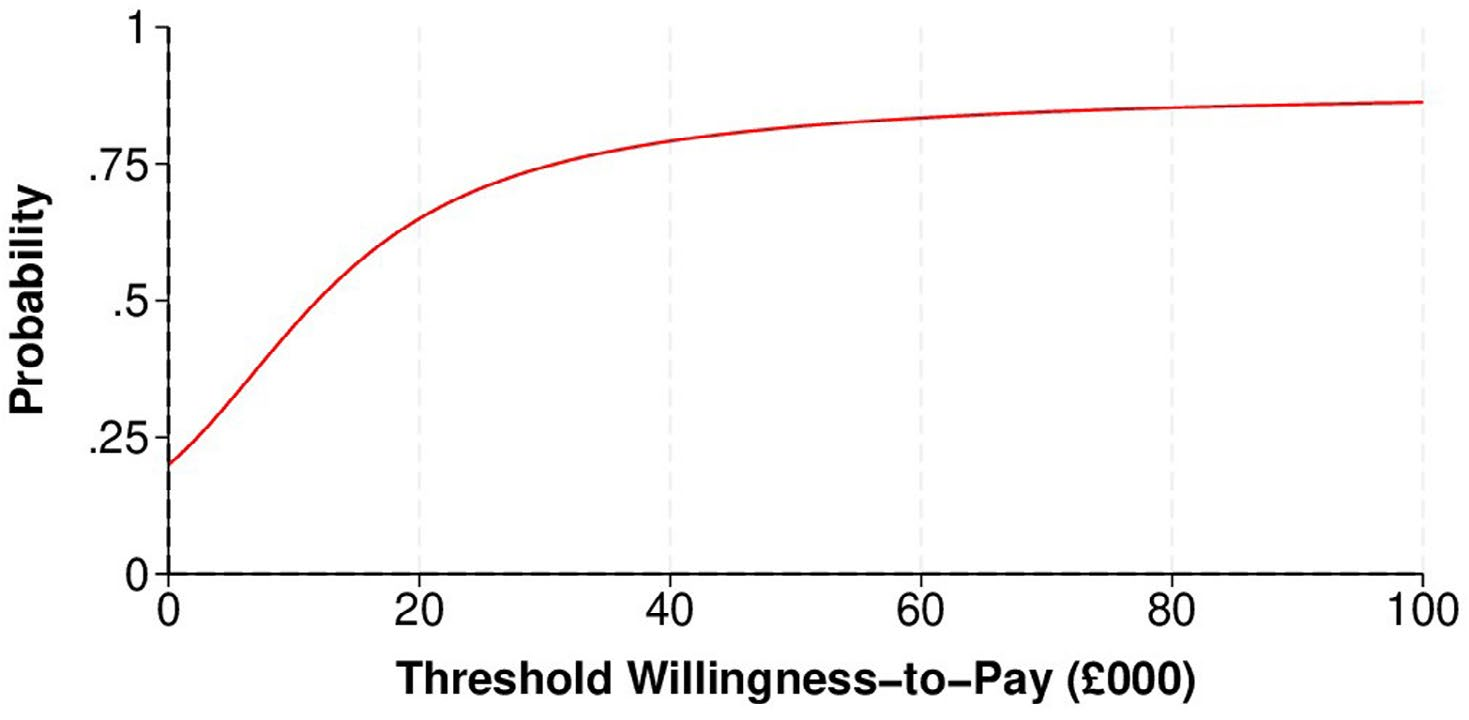
Cost‐effectiveness acceptability curve at 6 months for base‐case analysis of cases of prevented shoulder dystocia (NHS and PSS perspective, imputed data).

The early induction arm showed a lower incidence of shoulder dystocia compared to the standard care arm ((44/1439) and (33/1445), respectively), but the difference was not statistically significant (RR: 0.75%, 95% CI: −0.45% to 1.95%). However, the mean adjusted cost for the early induction arm was higher than for the standard care arm (MD: £89.38, 95% CI: −£78.64 to £257.40) over the entire follow‐up period. The incremental cost per case of shoulder dystocia prevented was £11 879. Considering hypothetical cost‐effectiveness thresholds of £5000, £10 000 and £20 000 for the prevention of a case of shoulder dystocia, the probabilities of early induction being cost‐effective were 0.32, 0.45, and 0.65, respectively (Table 2 and Figure 1, Appendix S1: Figures S1 and S2).

**TABLE 2:** Cost‐effectiveness: Incremental cost per case of prevented shoulder dystocia (2021–2022 prices), early induction compared with standard care.

Scenario	Treatment arm, mean (SE) cost (£)				Incremental cost (£)	Treatment arm, mean (SE) prevented shoulder dystocia		Incremental cases prevented shoulder dystocia (95% CI)	ICER (£)	Probability cost‐effective at			NMBs		
	*N*	Early induction	*N*	Standard care	(95% CI)	Early induction	Standard care			£5 K	£10 K	£20 K	£5 K	£10 K	£20 K
Base‐case analysis															
Imputed attributable cost and cases of prevented shoulder dystocia; covariate‐adjusted	1447	5490.16 (77.31)	1445	5400.78 (74.48)	89.38 (−78.64 to 257.40)	0.9771 (0.0042)	0.9695 (0.0042)	0.0075 (−0.0048 to 0.0117)	11 879	0.32	0.45	0.65	−52.00 (−267.00 to 163.00)	−14.00 (−251.00 to 223.00)	61.00 (−251.00 to 373.00)
Sensitivity analysis															
Complete case attributable cost and cases of prevented shoulder dystocia; covariate‐adjusted	403	5470.41 (116.44)	302	5144.13 (134.52)	326.28 (−5.43 to 657.99)	0.9775 (0.0083)	0.9638 (0.0095)	0.0137 (−0.0116 to 0.0391)	23 750	0.02	0.12	0.42	−258.00 (−498.00 to 17.00)	−189.00 (−510.00 to 132.00)	−52.00 (−586.00 to 483.00)
Complete case attributable cost and cases of prevented shoulder dystocia‐ bivariate, unadjusted	403	5472.60 (130.87)	302	5141.22 (108.48)	331.38 (−1.67 to 664.44)	0.9777 (0.0074)	0.9636 (0.0108)	0.0141 (−0.0114 to 0.0395)	23 517	0.02	0.12	0.43	−261.00 (−501.00 to 20.00)	−190.00 (−512.00 to 131.00)	−50.00 (−584.00 to 485.00)
Imputed attributable cost and QALYs; covariate‐adjusted‐modified ITT^a^	1445	5490.32 (75.54)	1439	5397.69 (70.89)	92.63 (−78.58 to 263.85)	0.9771 (0.0042)	0.9694 (0.0042)	0.0077 (−0.0041 to 0.0197)	11 904	0.31	0.45	0.65	−54.00 (−264.00 to 157.00)	−15.00 (−248.00 to 219.00)	63.00 (−247.00 to 377.00)
Per‐protocol analysis															
Clinical Estimand 2 analysis; imputed attributable cost and cases of prevented shoulder dystocia; covariate‐adjusted	1180	5509.54 (78.62)	1074	5328.60 (79.15)	180.94 (−5.08 to 366.97)	0.9771 (0.0049)	0.9626 (0.0052)	0.0145 (0.0005 to 0.0286)	12 479	0.18	0.39	0.73	−108 (−340 to 123)	−36 (−297 to 225)	109 (−247 to 465)

^a^ Modified ITT: Intention‐to‐treat (ITT) was modified by excluding eight participants with missing primary outcome (prevented shoulder dystocia).

In the early induction arm, women experienced a statistically non‐significant adjusted QALY gain (MD: 0.0023, 95% CI: −0.0005 to 0.0051) over the follow‐up period. The incremental cost per maternal QALY gained was £39 518, suggesting that early induction was not cost‐effective at the £20 000 and £30 000 cost‐effectiveness thresholds. The probability that early induction is cost‐effective was 0.36 and 0.44 at these thresholds, respectively. Additionally, the mean NMBs associated with early induction were negative (Table 3 and Appendix S1: Figures S3–S5).

**TABLE 3:** Cost‐effectiveness: Incremental cost per maternal QALY gained (2021–2022 prices), early induction compared with standard care.

Scenario	Treatment arm, mean (SE) cost (£)				Incremental cost (£)	Treatment arm, mean (SE) QALY		Incremental QALYs	ICER (£)	Probability cost‐effective at		NMBs	
	*N*	Early induction	*N*	Standard care	(95% CI)	Early induction	Standard care	(95% CI)		£20 K	£30 K	£20 K	£30 K
Base‐case analysis													
Imputed attributable cost and QALYs; covariate‐adjusted	1447	5490.16 (77.31)	1445	5400.78 (74.48)	89.38 (−78.64 to 257.40)	0.4308 (0.0015)	0.4285 (0.0017)	0.0023 (−0.0005 to 0.0051)	39 518	0.36	0.44	−44.00 (−283.00 to 195.00)	−22.00 (−285.00 to 242.00)
Sensitivity analysis													
Complete case attributable cost and QALYs; covariate‐adjusted	403	5470.41 (116.44)	302	5144.13 (134.52)	326.28 (−5.43 to 657.99)	0.4353 (0.0023)	0.4324 (0.0027)	0.0029 (−0.0041 to 0.0098)	113 400	< 0.01	0.002	−268.73 (−435.00 to −102.00)	−239.96 (−476.00 to −4.00)
Complete case attributable cost and QALYs ‐ bivariate, unadjusted	403	5472.60 (130.87)	302	5141.22 (108.48)	331.38 (−18.06 to 680.83)	0.4338 (0.0029)	0.4344 (0.0033)	−0.0007 (−0.0090 to 0.0077)	Standard care is dominant	0.01	< 0.01	−344.58 (−546.00 to −143.00)	−351.18 (−639.00 to −63.00)
Imputed attributable cost and QALYs (using a Hout crosswalk algorithm); covariate‐adjusted	1447	5498.64 (78.85)	1445	5396.10 (72.11)	102.54 (−64.81 to 269.88)	0.4304 (0.0016)	0.4332 (0.0016)	0.0028 (−0.0003 to 0.0052)	36 922	0.34	0.44	−47.00 (−277.00 to 183.00)	−19.00 (−269.00 to 230.00)
Imputed attributable cost and QALYs; covariate‐adjusted‐modified ITT^a^	1445	5489.31 (75.49)	1439	5395.73 (70.58)	93.58 (−77.34 to 264.50)	0.4309 (0.0016)	0.4286 (0.0016)	0.0024 (−0.0006 to 0.0053)	39 482	0.36	0.44	−44.00 (−283.00 to 195.00)	−22.00 (−285.00 to 242.00)
Per‐protocol analysis													
Clinical Estimand 2 analysis; imputed attributable cost and QALYs; covariate‐adjusted	1180	5509.54 (78.62)	1074	5328.60 (79.15)	180.94 (−5.08 to 366.97)	0.4311 (0.0017)	0.4296 (0.0019)	0.0016 (−0.0016 to 0.0047)	114 628	0.13	0.18	−149 (−405 to 106)	−134 (−419 to 152)

^a^ Modified ITT: Intention‐to‐treat (ITT) was modified by excluding eight participants with missing primary outcome (prevented shoulder dystocia).

### 3.6 Sensitivity, Per‐Protocol, and Subgroup Analyses

The sensitivity analyses based on complete cases only, estimating QALYs using the van Hout crosswalk algorithm, the modified ITT, and the per‐protocol analysis, supported the base‐case findings that early induction was associated with a trend towards higher mean costs, a slight increase in mean maternal QALYs, and a reduction in the incidence of shoulder dystocia (Tables 2 and 3).

Subgroup analyses showed that the mean incremental cost per case of shoulder dystocia prevented ranged from £4618 to £24 032 for participants with fetal weight centile ≤ 95% and a BMI ≥ 25 (Appendix S2: Table S5). Only two subgroup analyses suggested that the mean incremental cost per maternal QALY gained fell below a cost‐effectiveness threshold of £20 000: a fetal weight centile ≤ 95% (£5953; NMB: 0.69) and women with BMI ≥ 25 (£10 584; NMB: 0.54) (Appendix S2: Table S6).

### 3.7 Additional Analysis

The breakdown of costs and utility scores for planned caesarean compared to early induction and standard care are presented in Appendix S2: Tables S7–S10. Data from 274 women opting for planned caesarean section showed lower costs compared to both early induction and standard care, with mean differences of £310.00 (95% CI: −£545.21 to −£74.80) and £212.60 (95% CI: −£432.71 to £7.51), respectively. As shoulder dystocia cannot occur during a caesarean birth, in terms of incremental cost per case of shoulder dystocia prevented, caesarean section dominates both early induction and standard care. Maternal QALYs were higher in the early induction and standard care arms than in the caesarean section group, with mean differences of 0.0066 (95% CI: −0.0129 to 0.0001) and 0.0052 (95% CI: −0.0115 to 0.0011) maternal QALYs, respectively. Therefore, when we compared caesarean section, the incremental cost per maternal QALY gained was £46 775 and £41 184 for early induction and standard care respectively.

## 4 Discussion

This trial‐based economic evaluation of early induction of labour to prevent shoulder dystocia in suspected LGA foetuses showed a reduction in shoulder dystocia incidence but increased neonatal costs. Early induction had a 32%–65% probability of cost‐effectiveness across cost‐effectiveness thresholds of £5000–£20 000 per case of shoulder dystocia prevented. There was only a 36% probability of cost‐effectiveness at a threshold of £20 000 per maternal QALY gained. A per‐protocol analysis (Clinical Estimand 2) confirmed these results. The extensive sensitivity analyses also supported our base‐case findings. The within‐trial health economic evaluation therefore suggests that inducing labour for suspected LGA foetuses is unlikely to be cost‐effective. This analysis, covering follow‐up to 6 months postpartum, captured the immediate delivery effects but did not include potential long‐term impacts on infant and childhood QALYs. These could involve birth injuries from shoulder dystocia and subtle neurodevelopmental effects from early‐term deliveries [27].

Surprisingly, mean total costs were lower for women in the early induction arm, possibly due to fewer emergency caesarean sections. This difference may also be influenced by how unit costs were applied to each resource item. Our costing approach utilised the National Tariff method [15], which incorporates six payment levels for the different types of delivery and takes into account factors like induction, epidural, postpartum surgical intervention, and complication and comorbidity (CC) scores (Appendix S2: Table S1).

Combining costs for both mother and infant showed that early induction of labour led to higher NHS and PSS costs. This was primarily due to increased neonatal care costs immediately post‐birth and higher inpatient and outpatient care costs during the first 2 months postpartum. A 2005 decision modelling study in the USA supports these findings [10].

Our findings also suggest that choosing planned caesarean section is a less costly option compared to induction at 38 + 0 to 38 + 4 weeks of gestation. Whilst planned caesarean section prevents shoulder dystocia, it is associated with worse maternal HRQoL than early induction of labour and standard care. Therefore, when comparing caesarean section with early induction and standard care, the incremental cost per maternal QALY gained was not cost effective at £46 775 and £41 184, respectively. In addition, we did not assess the longer‐term consequences of caesarean sections, such as the increased risk of caesarean section in subsequent pregnancies. The results from this element of the study may also be prone to selection biases associated with observational research.

## 5 Strengths

Our research enabled us to conduct two pre‐defined primary analyses by addressing effects on the incidence of shoulder dystocia, maternal and infant costs, and maternal QALYs. The incidence of shoulder dystocia functions as a tangible clinical endpoint. However, the effect of this reduction on maternal and infant outcomes is not known. The health economic evaluation is based on an unbiased assessment of the consequences of early induction of labour in the context of the highly trained NHS labour ward workforce. This has led to findings that are counter to clinical expectations. Induction of labour to reduce the incidence of shoulder dystocia increased the cost of early infant care, with very small changes in maternal QALYs.

## 6 Limitations

Our analyses do not include QALY estimates for infants or long‐term health and social care costs beyond the trial follow‐up period. We also did not consider the long‐term costs associated with rare but serious complications of shoulder dystocia, such as permanent brachial plexus injuries and hypoxic ischaemic encephalopathy. Additionally, the potential impact of subtle long‐term neurodevelopmental differences from earlier delivery at term was not accounted for. However, it is noteworthy that two cases of hypoxic ischaemic encephalopathy occurred in the induction arm, and none in the standard care arm, and there were no fractures or permanent brachial plexus injuries following shoulder dystocia in the trial [7]. A neurodevelopmental follow‐up is ongoing at 2 years of age to assess potential harm from early induction. Additionally, the base‐case analysis utilised imputed data due to missing responses; only 705 out of 2893 total participants completed all resource use and EQ‐5D‐5L questions.

Our analysis was limited to an NHS and PSS perspective, omitting broader societal costs like economic losses from work and family expenses. We have also not considered the impact on partners/friends/family members, who might have had to take time off work or assume responsibilities for other dependents, including caring for children.

Caution is advised in interpreting the results as the small differences in QALYs led in some cases to substantial ICERs. Additionally, since no specific cost‐effectiveness threshold was identified in the literature for preventing cases of shoulder dystocia, we applied three hypothetical cost‐effectiveness thresholds to assess this outcome.

## 7 Conclusion

In conclusion, this economic evaluation indicates that early induction of labour for suspected LGA foetuses is associated with higher NHS and PSS costs for infants during the first 2 months postpartum. Early induction did not prove cost‐effective based on estimates of incremental cost per maternal QALY gained. It is important to note that these findings are constrained by the omission of long‐term costs and the omission of childhood QALYs.

## Author Contributions

S.Q., J.G., M.U., S.D. and S.P. designed the trial and the trial‐based economic evaluation. L.E., with support from Q.S., J.G., S.P., H.M. and M.U. led on developing data collection processes for these analyses. S.N. led the data analysis, with support from M.U., S.P., H.M. and S.D., S.N. and H.M. wrote the first draft of the manuscript, which all authors revised. All authors reviewed and agreed with the final version. All authors had access to all the data in the study and had final responsibility for the decision to submit for publication.

## Ethics Statement

This economic evaluation is a part of the Big Baby trial, which was conducted in accordance with the World Medical Association Declaration of Helsinki and applicable research governance standards. The study was reviewed and granted a favourable opinion on 19 March 2018 by the Southwest–Exeter Research Ethics Committee, Reference 18/SW/0039.

## Conflicts of Interest

Martin Underwood is the chief investigator or co‐investigator on multiple previous and current research grants from the UK National Institute for Health Research and is a co‐investigator on grants funded by the Australian NHMRC and Norwegian MRC. He is a director and shareholder of Clinvivo Ltd., which provides electronic data collection for health services research. He receives some salary support from University Hospitals Coventry and Warwickshire. He is a co‐investigator on two current and one completed NIHR‐funded studies that have, or have had, additional support from Stryker Ltd. Hema Mistry is a member of the NIHR HTA General Funding Commissioning Committee. Jason Gardosi is one of the two chief investigators of the Big Baby trial. He is the executive director of the Perinatal Institute, a not‐for‐profit social enterprise which has developed the GROW customised growth chart and centile calculator used in this study. The GROW software is licensed to most NHS hospitals as part of the Institute's national GAP programme of training, audit and software support, and 106 of these hospitals constituted the trial sites for the Big Baby Trial. Stavros Petrou receives support as a UK National Institute for Health Research (NIHR) Senior Investigator (NF‐SI‐0616‐10 103) and from the NIHR Applied Research Collaboration Oxford and Thames Valley.

## Supporting information


Appendix S1

Appendix S2

## Data Availability

The datasets used and/or used during the current study are available upon reasonable request from the WCTU data sharing committee (wctudataaccess@warwick.ac.uk).
