# Supplementary material for: Is It Cost‐Effective to Induce Labour Early to Prevent Shoulder Dystocia? Evidence From the Big Baby Trial
Source: BJOG. 2025 May 1;132(9):1250–8. doi: 10.1111/1471-0528.18160 (PMC12232589; doi:10.1111/1471-0528.18160)
Supplement: Supplementary file 1 — Appendix S1 [file BJO-132-1250-s002.docx]

**Table S1: Big Baby trial; NHS and PSS costs for participants with complete resource use data by treatment arm, study period, and cost category (2021–22 prices)**

| **Cost description** | **Adjusted^1^** | | | |
| --- | --- | --- | --- | --- |
|  | **Treatment arm, mean (SE^2^) cost (£)** | | **Between-arm differences**  **(95% CI)** | **p-value** |
|  | **Early induction (n=1,447)** | **Standard care**  **(n=1,445)** |  |  |
| Participants with complete data | N=403 | N= 302 |  | |
| **Woman’s cost** | | | | |
| **Antenatal phase** | | | | |
| Unscheduled hospital visit | 14.47 (3.39) | 42.26 (3.92) | -27.79  (-37.98 to -17.61) | <0.01 |
| Inpatient care | 5.06 (3.77) | 16.74 (4.35) | -11.68  (-22.99 to -0.37) | 0.04 |
| Medications | 0.51 (0.41) | 0.85 (0.48) | -0.34  (-1.58 to 0.89) | 0.59 |
| Total antenatal cost | 20.04 (4.29) | 59.86 (6.12) | -39.82  (-55.70 to -23.93) | <0.01 |
| **Delivery cost** | 3,481 (49.91) | 3,429 (57.65) | 52.01  (-97.72 to 201.75) | 0.49 |
| **Postnatal cost- between delivery and two months follow-up** | | | | |
| Hospital readmission within 30 days of postnatal inpatient discharge | 55.98 (13.62) | 56.74 (15.74) | -0.76  (-41.63 to 40.11) | 0.97 |
| Inpatient care | 17.74 (12.20) | 41.49 (14.09) | -23.75  (-60.35 to 12.83) | 0.20 |
| Outpatient care | 36.63 (4.52) | 28.32 (5.22) | 8.31  (-5.25 to 21.88) | 0.23 |
| Community care | 60.96 (3.37) | 55.70 (3.90) | 5.25  (-4.87 to 15.37) | 0.31 |
| Medications | 5.95 (0.81) | 4.39 (0.94) | 1.55  (-0.89 to 3.99) | 0.21 |
| Total cost at two months follow-up | 177.25 (24.45) | 187.24 (28.24) | -9.99  (-83.33 to 63.35) | 0.79 |
| **Postnatal cost- between two- and six-months follow-up** | | | | |
| Inpatient care | 4.68 (5.71) | 16.05 (6.60) | -11.37  (-28.52 to 5.77) | 0.19 |
| Outpatient care | 31.97 (4.99) | 34.13 (5.77) | -2.16  (-17.15 to 12.82) | 0.78 |
| Community care | 47.51 (3.38) | 43.35 (3.91) | 4.16  (-6.00 to 14.31) | 0.42 |
| Medications | 5.28 (0.84) | 2.81 (0.98) | 2.48  (-0.06 to 5.01) | 0.06 |
| Total cost at six months follow-up | 89.44 (9.23) | 96.34 (10.67) | -6.90  (-34.60 to 20.80) | 0.62 |
| Woman’s total cost | 3,768.49 (58.30) | 3,773.19 (67.34) | -4.70  (-179.59 to 170.19) | 0.96 |
| **Infant’s cost** | | | | |
| **Infant’s postpartum** (additional care in the same hospital where they were born, transferred to a different hospital and additional care in a different hospital) | | | | |
| Inpatient critical care^3^ | 861.23 (66.70) | 709.26 (77.04) | 151.97  (-48.13 to 352.07) | 0.14 |
| Transfer costs to a different hospital | 3.98 (6.92) | 22.12 (8.00) | -18.15  (-38.91 to 2.62) | 0.09 |
| Inpatient critical care in the different hospital | 12.20 (14.54) | 19.87 (16.80) | -7.67  (-51.30 to 35.96) | 0.73 |
| Total infant’s postpartum | 877.41 (68.75) | 751.25 (79.43) | 126.15  (-80.12 to 332.42) | 0.23 |
| **Infant’s two months follow-up** | | | | |
| Hospital readmission within 30 days of postnatal inpatient discharge | 129.54 (27.33) | 33.62 (31.57) | 95.93  (13.94 to 177.91) | 0.02 |
| Inpatient care | 123.49 (35.85) | 67.99 (41.41) | 55.50  (-52.05 to 163.05) | 0.31 |
| Outpatient care | 131.72 (9.53) | 85.10 (11.01) | 46.61  (18.03 to 75.20) | <0.001 |
| Community care | 213.99 (5.16) | 209.40 (5.96) | 4.59 (-10.90 to 20.08) | 0.56 |
| Medications | 5.38 (1.30) | 3.49 (1.50) | 1.89 (-2.00 to 5.78) | 0.34 |
| Antibiotic | 0.50 (0.13) | 0.34 (0.15) | 0.15 (-0.23 to 0.53) | 0.43 |
| Infant’s total cost at two months | 605.04 (61.42) | 399.94 (70.95) | 205.10  (20.84 to 389.36) | 0.03 |
| **Infant’s six months follow-up** | | | | |
| Inpatient care | 26.83 (14.26) | 42.16 (16.48) | -15.33  (-58.13 to 27.46) | 0.48 |
| Outpatient care | 80.08 (8.59) | 66.68 (9.91) | 13.40  (-12.34 to 39.14) | 0.31 |
| Community care | 101.17 (4.57) | 104.56 (5.27) | -3.38  (-17.08 to 10.32) | 0.63 |
| Medications | 10.66 (3.41) | 5.62 (3.93) | 5.04 (-5.17 to 15.26) | 0.33 |
| Antibiotic | 0.73 (0.19) | 0.72 (0.22) | 0.00 (-0.58 to 0.58) | 0.99 |
| Infant’s total cost at six months | 219.47 (19.66) | 219.74 (22.71) | -0.27  (-59.26 to 58.71) | 0.99 |
| Infant’s total cost | 1,701.92 (96.18) | 1,370.94 (111.10) | 330.98  (42.44 to 619.52) | 0.02 |
| **Combined, woman and infant’s cost** | | | | |
| Intrapartum and postnatal period | 4,359.16 (87.85) | 4,181.00 (101.48) | 178.16  (-85.39 to 441.72) | 0.18 |
| Two months | 782.30 (66.79) | 587.19 (77.15) | 195.11  (-5.26 to 395.48) | 0.06 |
| Six months | 308.91 (22.74) | 316.08 (26.28) | -7.17  (-75.42 to 61.07) | 0.84 |
| Total cost | 5,470.41 (116.45) | 5,144.13 (134.52) | 326.28  (-23.07 to 675.63) | 0.07 |

^1^ Adjusted with maternal age, fetal weight centile, and recruitment site.

^2^ SE, Standard error

^3^ Inpatient critical care mentions to critical care costs rather than the infants being critically ill.


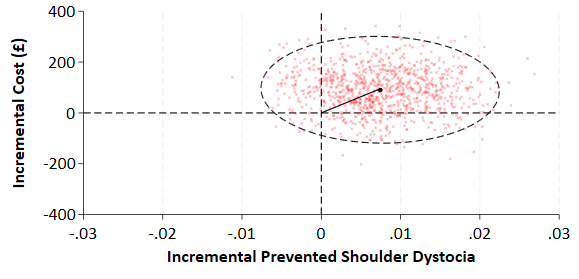


**Figure S1: Cost-effectiveness scatterplot at six months for base case analysis of case of prevented shoulder dystocia (NHS and PSS perspective, Imputed data).**


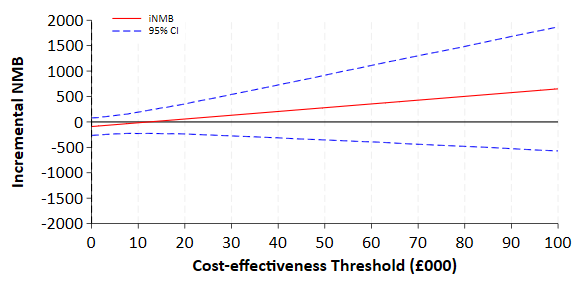


**Figure S2: Net monetary benefit (NMB) at six months for base case analysis of case of prevented shoulder dystocia (NHS and PSS perspective, Imputed data).**


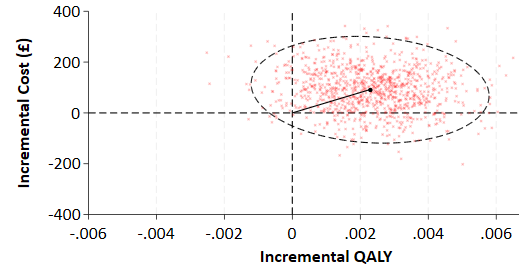


**Figure S3: Cost-effectiveness scatterplot at six months for base case analysis; maternal QALY gained (NHS and PSS perspective, imputed data).**


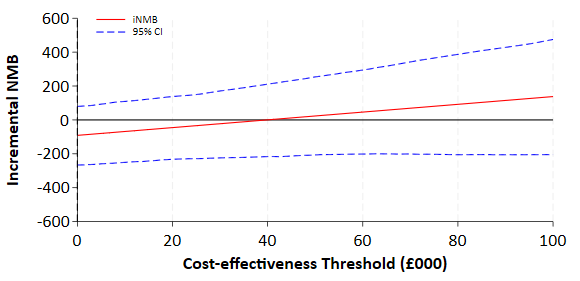


**Figure S4: Net monetary benefit (NMB) at six months for base case analysis; maternal QALY gained (NHS and PSS perspective, imputed data).**


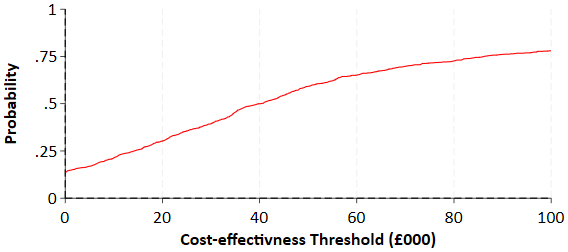


**Figure S5: Cost-effectiveness acceptability curve at six months for base case analysis; maternal QALY gained (NHS and PSS perspective, imputed data).**
