# Supplementary material for: Is It Cost‐Effective to Induce Labour Early to Prevent Shoulder Dystocia? Evidence From the Big Baby Trial
Source: BJOG. 2025 May 1;132(9):1250–8. doi: 10.1111/1471-0528.18160 (PMC12232589; doi:10.1111/1471-0528.18160)
Supplement: Supplementary file 2 — Appendix S2 [file BJO-132-1250-s001.docx]

***Appendix***

***Table A1: Unit costs associated with woman and infant’s resource use items***

| **Infant Inpatient; Source: National schedule of NHS costs - Year 2021/22 (£)**^18^ | | | | | | | | | |
| --- | --- | --- | --- | --- | --- | --- | --- | --- | --- |
| **HRG code** | **Currency description** | | | | **Unit cost per day** | | | | |
| XA01Z | Neonatal Critical Care, Intensive Care | | | | 1,819 | | | | |
| XA02Z | Neonatal Critical Care, High Dependency | | | | 1,231 | | | | |
| XA03Z | Neonatal Critical Care, Special Care, without External Carer | | | | 935 | | | | |
| XA04Z | Neonatal Critical Care, Special Care, with External Carer | | | | 658 | | | | |
| XA05Z | Neonatal Critical Care, Neonatology Supported Care | | | | 684 | | | | |
| XA06Z | Neonatal Critical Care, Transportation | | | | 1,657 | | | | |
| XB07Z | Paediatric Critical Care, Basic Critical Care | | | | 1,645 | | | | |
| XB06Z | Paediatric Critical Care, Intermediate Critical Care | | | | 2,166 | | | | |
| PHCD00404 | High-Cost Drug during inpatient stay: Nitric Oxide | | | | 1,884.90 | | | | |
| **HRG code** | **Currency description** | | | | **Day case** | **Non-elective-Short Stay** | | | **Non-elective-Long Stay** |
| PB06M | Neonatal Diagnoses, Admitted from Home, without Interventions, with CC Score 0 | | | | 569 | 593 | | | 1,915 |
| PX57C | Paediatric, Examination, Follow-up, Special Screening or Other Admissions, with CC Score 0 | | | | 656 | 645 | | | 1,921 |
| PF26C | Paediatric Other Gastrointestinal Disorders with CC Score 0 | | | | 619 | 565 | | | 2,513 |
| PD11C | Paediatric, Acute Upper Respiratory Tract Infection or Common Cold, with CC Score 0 | | | | 459 | 490 | | | 1,250 |
| PD14F | Paediatric Lower Respiratory Tract Disorders without Acute Bronchiolitis, with CC Score 0 | | | | 552 | 744 | | | 2,608 |
| PE62C | Paediatric, Syncope and Collapse, with CC Score 0 | | | | 750 | 477 | | | 1,295 |
| PJ66C | Paediatric, Rash or Other Non-Specific Skin Eruption, with CC Score 0 | | | | 517 | 487 | | | 1,541 |
| PD15D | Paediatric Acute Bronchiolitis with CC Score 0 | | | | 447 | 666 | | | 2,944 |
| PF21B | Paediatric, Infectious or Non-Infectious Gastroenteritis, with CC Score 0 | | | | 543 | 549 | | | 1,791 |
| PF28E | Paediatric, Feeding Difficulties or Vomiting, with CC Score 0 | | | | 563 | 569 | | | 2,237 |
| PW20C | Paediatric Fever of Unknown Origin with CC Score 0 | | | | 471 | 552 | | | 2,345 |
| PW01C | Paediatric Minor Infections with CC Score 0 | | | | 565 | 513 | | | 1,685 |
| PF27B | Paediatric Inflammatory Bowel Disease with CC Score 0 | | | | 562 | 698 | | | 4,022 |
| PW17D | Paediatric Intermediate Infections with CC Score 0 | | | | 522 | 615 | | | 2,750 |
| PX50C | Paediatric, Ingestion Poisoning or Allergies, with CC Score 0 | | | | 615 | 663 | | | 1,798 |
| PX30C | Paediatric Faltering Growth (Failure to Thrive) with CC Score 0 | | | | 764 | 637 | | | 2,832 |
| PD12C | Paediatric, Asthma or Wheezing, with CC Score 0 | | | | 566 | 774 | | | 2,141 |
| DX21B | COVID-19 Infection, 18 years and under | | | | 501 | 836 | | | 3,153 |
| PE24C | Paediatric, Arrhythmia or Conduction Disorders, with CC Score 0 | | | | 996 | 647 | | | 2,650 |
| PC63D | Paediatric, Head, Neck or Ear Disorders, with CC Score 0 | | | | 743 | 567 | | | 1,896 |
| PL70D | Paediatric Other Renal Diseases with CC Score 0 | | | | 587 | 611 | | | 2,073 |
| PR06C | Paediatric Intracranial Injury with CC Score 0 | | | | 525 | 1,037 | | | 2,654 |
| PR01E | Paediatric Nervous System Disorders with CC Score 0 | | | | 1,248 | 635 | | | 2,561 |
| PE23F | Paediatric Cardiac Conditions with CC Score 0 | | | | 966 | 716 | | | 2,461 |
| PH34D | Paediatric, Musculoskeletal or Connective Tissue Disorders, with CC Score 0 | | | | 734 | 599 | | | 2,854 |
| PW16E | Paediatric Major Infections with CC Score 0 | | | | 696 | 838 | | | 4,252 |
| PL38C | Paediatric Renal Disease with Renal Failure, with CC Score 0 | | | | 669 | 741 | | | 3,351 |
| PR03C | Paediatric Febrile Convulsions with CC Score 0 | | | | 833 | 666 | | | 2,333 |
| PJ35D | Paediatric Skin Disorders with CC Score 0 | | | | 709 | 538 | | | 2,519 |
| PK72C | Paediatric Metabolic Disorders with CC Score 0 | | | | 1,004 | 965 | | | 6,667 |
| CA65Z | Frenotomy or Frenectomy | | | | 989 | 1,121 | | | 1,867 |
| PP64B | Paediatric Non-Surgical Ophthalmology with CC Score 0 | | | | 776 | 650 | | | 2,937 |
| FF62F | Inguinal, Umbilical or Femoral Hernia Procedures, 1 year and under | | | | 2,764 | 3,185 | | | 7,106 |
| JB60B | Treatment of Unspecified Degree of Burn, 15 years and under | | | | 1,386 | 1,041 | | | 5,879 |
| DZ26P | Pneumothorax or Intrathoracic Injuries, without Interventions, with CC Score 0-2 | | | | 274 | 504 | | | 2,182 |
| JC43D | Minor Skin Procedures, 18 years and under | | | | 1,429 | 1,268 | | | 1,129 |
| LB55B | Minor or Intermediate, Urethra Procedures, 18 years and under | | | | 1,746 | 2,743 | | | 2,554 |
| HN45C | Minor Hand Procedures for Non-Trauma, 5 years and under | | | | 1,855 | 1,867 | | | - |
| Infant’s Outpatient care; Source: National schedule of NHS costs - Year 2021/22 (£)^18^ | | | | | | | | | |
| **HRG code** | **Currency description** | | | | **Unit cost per day** | | | | |
| CA65Z | Frenotomy or Frenectomy | | | | 265 | | | | |
| CD05B | Surgical Removal of Tooth, 18 years and under | | | | 321 | | | | |
| Infant Outpatient care and Procedure; Source: National tariff- Year 2022/23 (version of April 2022) (£)^17^ | | | | | | | | | |
| **code** | **Treatment function description** | | | | **WF01B  First Attendance** | | **WF01A  Follow Up Attendance** | | |
| 420 | Paediatric Service | | | | 232 | | 156 | | |
| 650 | Physiotherapy Service | | | | 100 | | 100 | | |
| 171 | Paediatric Surgery Service | | | | 191 | | 115 | | |
| 211 | Paediatric Urology Service | | | | 145 | | 86 | | |
| 214 | Paediatric Trauma and Orthopaedic Service | | | | 169 | | 94 | | |
| 215 | Paediatric Ear Nose and Throat Service | | | | 127 | | 68 | | |
| 216 | Paediatric Ophthalmology Service | | | | 139 | | 82 | | |
| 219 | Paediatric Plastic Surgery Service | | | | 145 | | 96 | | |
| 251 | Paediatric Gastroenterology Service | | | | 279 | | 162 | | |
| 253 | Paediatric Clinical Haematology Service | | | | 400 | | 189 | | |
| 257 | Paediatric Dermatology Service | | | | 156 | | 104 | | |
| 258 | Paediatric Respiratory Medicine Service | | | | 278 | | 148 | | |
| 321 | Paediatric Cardiology Service | | | | 213 | | 131 | | |
| 800 | Clinical Oncology Service | | | | 284 | | 132 | | |
| VB01-9Z | Average of Emergency Medicine, Any Category, Any Investigation with Treatment (Type 1 and 2  Departments) | | | | 253.22 | | 253.22 | | |
| RD40-3Z | Average of Ultrasound Scan with duration of less and more than 20 minutes, with and without Contrast | | | | 70.5 | | 70.5 | | |
| RD01-2C | Average of Magnetic Resonance Imaging Scan of One Area, without Contrast and with Post-Contrast Only, 5 years and under | | | | 183.5 | | 183.5 | | |
| Women Outpatient care and Procedure; Source: National tariff- Year 2022/23 (version of April 2022) (£)^17^ | | | | | | | | | |
| **code** | **Treatment function description** | | | | **WF01B  First Attendance** | | **WF01A  Follow Up Attendance** | | |
| 501 | Obstetrics Service | | | | 170 | | 92 | | |
| 560 | Midwifery Service | | | | 201 | | 78 | | |
| 501&560 | Average 501 and 560 | | | | 186 | | 85 | | |
| 502 | Gynaecology Service | | | | 167 | | 78 | | |
| VB01Z-09Z | Average of Emergency Medicine, Any Investigation Any Category with Treatment (Type 1,2 and 3  Departments) | | | | 169.11 | | 169.11 | | |
| RD01-2A | Average of Magnetic Resonance Imaging Scan of One Area, with and without Contrast, 19 years and over | | | | 140 | | 140 | | |
| RN34A | Other Specified Diagnostic Imaging of Other Sites, 19 years and over | | | | 191 | | 191 | | |
| RD51A | Simple Echocardiogram, 19 years and over | | | | 82 | | 82 | | |
| RD20-1A | Average of Computerised Tomography Scan of One Area, with and without Post-Contrast Only, 19 years and over | | | | 77 | | 77 | | |
| 100 | General Surgery Service | | | | 181 | | 78 | | |
| 101 | Urology Service | | | | 145 | | 71 | | |
| 103 | Breast Surgery Service | | | | 187 | | 79 | | |
| 104 | Colorectal Surgery Service | | | | 157 | | 68 | | |
| 105 | Hepatobiliary and Pancreatic Surgery Service | | | | 269 | | 115 | | |
| 106 | Upper Gastrointestinal Surgery Service | | | | 187 | | 80 | | |
| 107 | Vascular Surgery Service | | | | 194 | | 85 | | |
| 108 | Spinal Surgery Service | | | | 222 | | 88 | | |
| 110 | Trauma and Orthopaedic Service | | | | 169 | | 67 | | |
| 120 | Ear Nose and Throat Service | | | | 133 | | 57 | | |
| 130 | Ophthalmology Service | | | | 142 | | 64 | | |
| 140 | Oral Surgery Service | | | | 142 | | 69 | | |
| 143 | Orthodontic Service | | | | 192 | | 76 | | |
| 170 | Cardiothoracic Surgery Service | | | | 286 | | 123 | | |
| 172 | Cardiac Surgery Service | | | | 286 | | 123 | | |
| 173 | Thoracic Surgery Service | | | | 303 | | 120 | | |
| 301 | Gastroenterology Service | | | | 215 | | 85 | | |
| 302 | Endocrinology Service | | | | 246 | | 99 | | |
| 303 | Clinical Haematology Service | | | | 288 | | 131 | | |
| 306 | Hepatology Service | | | | 291 | | 153 | | |
| 307 | Diabetes Service | | | | 134 | | 106 | | |
| 320 | Cardiology Service | | | | 179 | | 94 | | |
| 321 | Paediatric Cardiology Service | | | | 213 | | 131 | | |
| 329 | Transient Ischaemic Attack Service | | | | 402 | | - | | |
| 330 | Dermatology Service | | | | 134 | | 74 | | |
| 340 | Respiratory Medicine Service | | | | 225 | | 100 | | |
| 341 | Respiratory Physiology Service | | | | 171 | | 80 | | |
| 350 | Infectious Diseases Service | | | | 375 | | 154 | | |
| 361 | Renal Medicine Service | | | | 183 | | 133 | | |
| 370 | Medical Oncology Service | | | | 298 | | 135 | | |
| 410 | Rheumatology Service | | | | 283 | | 96 | | |
| 420 | Paediatric Service | | | | 232 | | 156 | | |
| 430 | Elderly Medicine Service | | | | 308 | | 145 | | |
| 502 | Gynaecology Service | | | | 167 | | 78 | | |
| 503 | Gynaecological Oncology Service | | | | 159 | | 77 | | |
| 800 | Clinical Oncology Service | | | | 284 | | 132 | | |
| Mothers Outpatient; Source: National schedule of NHS costs - Year 2021/22 (£)^18^ | | | | | | | | | |
| **Service code** | **Service description** | | | | **Unit cost** | | | | |
| 650 | Physiotherapy Service | | | | 100 | | | | |
| 724 | Perinatal Mental Health Service | | | | 180 | | | | |
| 710 | Adult Mental Health Service | | | | 295 | | | | |
| 400 | Neurology Service | | | | 214 | | | | |
| 401 | Clinical Neurophysiology Service | | | | 309 | | | | |
| Woman’s Inpatient; Source: National tariff workbook 2022/23, version April 2022- Maternity pathway^17^ | | | | | | | | | |
| **Delivery Phase- mapping HRGs to 6 payment levels** | | | | | | | | | |
| **HRG code** | **HRG description** | | | | **Unit cost (£)** | | **Ordinary trim-point** | | **Unit cost for days exceeding**  **time-point (£)** |
| NZ30C | Normal Delivery with CC Score 0 | | | | 2,014 | | 5 | | 464 |
| NZ30A | Normal Delivery with CC Score 2+ | | | | 2,455 | | 5 | | 464 |
| NZ30B | Normal Delivery with CC Score 1 | | | | 2,455 | | 5 | | 464 |
| NZ31C | Normal Delivery, with Epidural or Induction, with CC Score 0 | | | | 2,455 | | 5 | | 464 |
| NZ40C | Assisted Delivery with CC Score 0 | | | | 2,455 | | 5 | | 464 |
| NZ31A | Normal Delivery, with Epidural or Induction, with CC Score 2+ | | | | 3,275 | | 6 | | 464 |
| NZ31B | Normal Delivery, with Epidural or Induction, with CC Score 1 | | | | 3,275 | | 6 | | 464 |
| NZ32B | Normal Delivery, with Epidural and Induction, or with Post-Partum Surgical Intervention, with CC Score 1 | | | | 3,275 | | 6 | | 464 |
| NZ32C | Normal Delivery, with Epidural and Induction, or with Post-Partum Surgical Intervention, with CC Score 0 | | | | 3,275 | | 6 | | 464 |
| NZ33B | Normal Delivery, with Epidural or Induction, and with Post-Partum Surgical Intervention, with CC Score 1 | | | | 3,275 | | 6 | | 464 |
| NZ33C | Normal Delivery, with Epidural or Induction, and with Post-Partum Surgical Intervention, with CC Score 0 | | | | 3,275 | | 6 | | 464 |
| NZ34C | Normal Delivery, with Epidural, Induction and Post-Partum Surgical Intervention, with CC Score 0 | | | | 3,275 | | 6 | | 464 |
| NZ40A | Assisted Delivery with CC Score 2+ | | | | 3,275 | | 6 | | 464 |
| NZ40B | Assisted Delivery with CC Score 1 | | | | 3,275 | | 6 | | 464 |
| NZ41B | Assisted Delivery, with Epidural or Induction, with CC Score 1 | | | | 3,275 | | 6 | | 464 |
| NZ41C | Assisted Delivery, with Epidural or Induction, with CC Score 0 | | | | 3,275 | | 6 | | 464 |
| NZ42C | Assisted Delivery, with Epidural and Induction, or with Post-Partum Surgical Intervention, with CC Score 0 | | | | 3,275 | | 6 | | 464 |
| NZ43C | Assisted Delivery, with Epidural or Induction, and with Post-Partum Surgical Intervention, with CC Score 0 | | | | 3,275 | | 6 | | 464 |
| NZ50C | Planned Caesarean Section with CC Score 0-1 | | | | 3,275 | | 6 | | 464 |
| NZ32A | Normal Delivery, with Epidural and Induction, or with Post-Partum Surgical Intervention, with CC Score 2+ | | | | 4,044 | | 7 | | 464 |
| NZ33A | Normal Delivery, with Epidural or Induction, and with Post-Partum Surgical Intervention, with CC Score 2+ | | | | 4,044 | | 7 | | 464 |
| NZ34A | Normal Delivery, with Epidural, Induction and Post-Partum Surgical Intervention, with CC Score 2+ | | | | 4,044 | | 7 | | 464 |
| NZ34B | Normal Delivery, with Epidural, Induction and Post-Partum Surgical Intervention, with CC Score 1 | | | | 4,044 | | 7 | | 464 |
| NZ42B | Assisted Delivery, with Epidural and Induction, or with Post-Partum Surgical Intervention, with CC Score 1 | | | | 4,044 | | 7 | | 464 |
| NZ43B | Assisted Delivery, with Epidural or Induction, and with Post-Partum Surgical Intervention, with CC Score 1 | | | | 4,044 | | 7 | | 464 |
| NZ41A | Assisted Delivery, with Epidural or Induction, with CC Score 2+ | | | | 4,044 | | 7 | | 464 |
| NZ44B | Assisted Delivery, with Epidural, Induction and Post-Partum Surgical Intervention, with CC Score 1 | | | | 4,044 | | 7 | | 464 |
| NZ44C | Assisted Delivery, with Epidural, Induction and Post-Partum Surgical Intervention, with CC Score 0 | | | | 4,044 | | 7 | | 464 |
| NZ50B | Planned Caesarean Section with CC Score 2-3 | | | | 4,044 | | 7 | | 464 |
| NZ42A | Assisted Delivery, with Epidural and Induction, or with Post-Partum Surgical Intervention, with CC Score 2+ | | | | 4,746 | | 10 | | 464 |
| NZ43A | Assisted Delivery, with Epidural or Induction, and with Post-Partum Surgical Intervention, with CC Score 2+ | | | | 4,746 | | 10 | | 464 |
| NZ44A | Assisted Delivery, with Epidural, Induction and Post-Partum Surgical Intervention, with CC Score 2+ | | | | 4,746 | | 10 | | 464 |
| NZ51C | Emergency Caesarean Section with CC Score 0-1 | | | | 4,746 | | 10 | | 464 |
| NZ51B | Emergency Caesarean Section with CC Score 2-3 | | | | 5,977 | | 13 | | 464 |
| NZ16Z | Ante-Natal Routine Observation | | | | 363 | | 5 | | 487 |
| NZ17A | Ante-Natal False Labour, including Premature Rupture of Membranes, with CC Score 2+ | | | | 776 | | 5 | | 487 |
| NZ17B | Ante-Natal False Labour, including Premature Rupture of Membranes, with CC Score 0-1 | | | | 576 | | 5 | | 487 |
| NZ18A | Ante-Natal Complex Disorders with CC Score 2+ | | | | 1,570 | | 5 | | 487 |
| NZ18B | Ante-Natal Complex Disorders with CC Score 0-1 | | | | 1,039 | | 5 | | 487 |
| NZ19A | Ante-Natal Major Disorders with CC Score 2+ | | | | 701 | | 5 | | 487 |
| NZ19B | Ante-Natal Major Disorders with CC Score 0-1 | | | | 507 | | 5 | | 487 |
| NZ20A | Ante-Natal Other Disorders with CC Score 2+ | | | | 556 | | 5 | | 487 |
| NZ20B | Ante-Natal Other Disorders with CC Score 0-1 | | | | 426 | | 5 | | 487 |
| NZ21Z | Ante-Natal Standard Routine Ultrasound Scan | | | | 113 | | 5 | | 487 |
| NZ22Z | Ante-Natal Specialised Non-Routine Ultrasound Scan | | | | 96 | | 5 | | 487 |
| NZ24A | Ante-Natal Therapeutic Procedures, including Induction, with CC Score 2+ | | | | 1,188 | | 5 | | 487 |
| NZ24B | Ante-Natal Therapeutic Procedures, including Induction, with CC Score 0-1 | | | | 758 | | 5 | | 487 |
| NZ25Z | Labour without Specified Delivery | | | | 1,584 | | 5 | | 487 |
| NZ26A | Post-Natal Disorders with CC Score 2+ | | | | 1,585 | | 8 | | 487 |
| NZ26B | Post-Natal Disorders with CC Score 0-1 | | | | 949 | | 5 | | 487 |
| NZ27Z | Post-Natal Therapeutic Procedures | | | | 1,902 | | 5 | | 487 |
| Woman’s Inpatient; Source: National schedule of NHS costs - Year 2021/22 (£)^18^ | | | | | | | | | |
| **HRG code** | **Currency description** | | | | **Day case** | | **Non-elective-Short Stay** | | **Non-elective-Long Stay** |
| CA85A | Minor, Mouth or Throat Procedures, 19 years and over | | | | 967 | | 790 | | 1,543 |
| DX21A | COVID-19 Infection, 19 years and over | | | | 300 | | 635 | | 3,278 |
| DZ11V | Lobar, Atypical or Viral Pneumonia, without Interventions, with CC Score 0-3 | | | | 325 | | 543 | | 2,068 |
| FF37D | Appendicectomy Procedures, 19 years and over, with CC Score 0 | | | | 3,146 | | 3,546 | | 4,976 |
| FF53A | Minor Therapeutic or Diagnostic, General Abdominal Procedures, 19 years and over | | | | 952 | | 1,046 | | 2,224 |
| GA10N | Open Cholecystectomy, 19 years and over, with CC Score 0 | | | | 3,651 | | 1,761 | | 7,200 |
| HE52F | Other Injury of Arm without Interventions, with CC Score 0-1 | | | | 584 | | 443 | | 1,620 |
| MA22Z | Minor Lower Genital Tract Procedures | | | | 1,775 | | 2,001 | | 1,875 |
| WD09Z | Other Mental Health Disorders, treated by a Non-Specialist Mental Health Service Provider | | | | 418 | | 574 | | 4,456 |
| WH05Z | Allergy or Adverse Allergic Reaction | | | | 320 | | 439 | | 1,071 |
| FF63A | Herniotomy Procedures, 2 years and over | | | | 2,120 | | 2,516 | | 7,279 |
| GA10N | Open Cholecystectomy, 19 years and over, with CC Score 0 | | | | 3,651 | | 1,761 | | 7,200 |
| HE31C | Foot Fracture with Single Intervention, with CC Score 0-1 | | | | - | | 1,074 | | 3,585 |
| Women and infants community care; Source: Unit Costs of Health and Social Care 2021/2022^19^ | | | | | | | | | |
| **Type of appointment** | | | **Description** | | **Unit Cost per hour (£)** | | | **Unit cost per visit (£)** | |
| GP surgery visit | | | Per surgery consultation lasting 9.22 minutes | | **-** | | | 42 | |
| GP out-of-hours | | | 10 minutes of Associate specialist and 20 minutes of Nurse, band 6 | | 137 and 53 | | | 40.5 | |
| Walk-in-centre | | | 10 minutes of Associate specialist and 20 minutes of Nurse, band 6 | | 137 and 53 | | | 40.5 | |
| Practice nurse | | | 30 minutes | | 52 | | | 26 | |
| Qualified nurse (midwife, health visitor) | | | 30 minutes | | 57 | | | 28.5 | |
| Nursery nurse | | | 30 minutes, band 4 | | 33 | | | 16.5 | |
| Nurse mental health | | | 30 minutes, band 5 | | 43 | | | 21.5 | |
| Nurse specialist | | | 30 minutes, band 6 | | 53 | | | 26.5 | |
| Consultant: medical | | | 15 minutes | | 148 | | | 37 | |
| Consultant: surgical | | | 15 minutes | | 142 | | | 35.5 | |
| Physiotherapist | | | 30 minutes, band 5 | | 42 | | | 21 | |
| Counsellor | | | 30 minutes, band 5 | | 42 | | | 21 | |
| Clinical psychologist | | | 30 minutes, band 5 | | 66 | | | 33 | |
| Pharmacist | | | 30 minutes, band 6 | | 55 | | | 27.5 | |
| Podiatrist | | | 30 minutes, band 5 | | 42 | | | 21 | |
| Chiropractor | | | 30 minutes, band 5 | | 42 | | | 21 | |
| Occupational therapist | | | 30 minutes, band 5 | | 42 | | | 21 | |
| Dentist | | | 30 minutes of average of NHS dentist – Performer-Only and Dentist – Providing-Performer | | 150 and 108 | | | 64.5 | |
| Telephone triage – GP-led and nurse-led | | | Five minutes of Nurse-led and GP-led | | 70 and 237 | | | 13.17 | |
| Social worker | | | 30 minutes | | 50 | | | 25 | |
| Support and outreach worker | | | 30 minutes | | 25 | | | 12.5 | |
| GP telephone calls | | | Average cost per e-consultation | | - | | | 14.43 | |
| Calculating the unit cost for other type of appointment according to the Personal Social Services Research Unit (PSSRU) Unit Costs of Health and Social Care manual 2022^19^ | | | | | | | | | |
| **Type of appointment** | | **Average wage (£)** | **salary oncost, Overhead-Management, admin and estates staff, non-staff (£)** | **Capital overheads (£)** | **Working time** | | | **cost per 30 min/ 15 min (£)** | |
| Audiologist | | 44,000 | 57,200 | 6,317 | 1,635 | | | 30.95 | |
| Optometrist | | 50,000 | 65,000 | 6,317 | 1,635 | | | 35.17 | |
| Dietitian | | 40,909 | 53,181.7 | 6,317 | 1,635 | | | 28.77 | |
| Nutritionist | | 37,000 | 48,100 | 6,317 | 1,635 | | | 26.02 | |
| Paramedic | | 42,000 | 54,600 | 6,317 | 1,635 | | | 29.54 | |
| Community Paediatrician | | £102,185 | 13,2840.5 | 6,317 | 1,635 | | | 35.94 | |

***Table A2: Number and proportion of individuals with missing health economic data by treatment arm***

| **Description** | **Treatment arm, missing values, n (%)** | | **Total missing values, n (%)** |
| --- | --- | --- | --- |
|  | **Early induction**  **(n=1,447)** | **Standard care**  **(n=1,445)** |  |
| **Outcomes** | | | |
| Primary: shoulder dystocia | 2 (0.1) | 6 (0.4) | 8 (0.3) |
| Secondary: utility | | | |
| EQ-5D index score at baseline | 14 (1) | 28 (2) | 42 (1) |
| EQ-5D index score at two months follow-up | 490 (34) | 633 (44) | 1,123 (39) |
| EQ-5D index score at six months follow-up | 673 (47) | 825 (57) | 1,498 (52) |
| QALYs generated from EQ-5D utility scores | 751 (51) | 911 (63) | 1,662 (57) |
| **Resources used** | | | |
| Woman’s resources used | | | |
| Resources used between delivery and two months | 600 (41) | 726 (50) | 1,326 (46) |
| Resources used between two and six months | 771 (53) | 909 (63) | 1,680 (58) |
| Resources used between delivery and six months | 886 (61) | 1,016 (70) | 1,902 (66) |
| Infant’s resources used | | | |
| Resources used between birth and two months | 637 (44) | 730 (51) | 1,367 (47) |
| Resources used between two and six months | 790 (55) | 920 (64) | 1,710 (59) |
| Resources used between birth and six months | 912 (63) | 1,030 (71) | 1,942 (67) |
| Combined woman and infant’s resources used (n: early induction = 2,894, standard care= 2,890) | | | |
| Resources used between delivery and two months | 1,237 (46) | 1,456 (50) | 2,693 (47) |
| Resources used between two and six months | 1,561 (54) | 1,829 (63) | 3,390 (59) |
| Resources used between delivery and six months | 1,798 (62) | 2,046 (71) | 3,844 (66) |

***Table A3: Use of health and social care resources by follow-up period and treatment arm (complete case)***

| **Resource** | **Induction of labour**  **(N=1,446)** | **Standard care**  **(N=1,445)** | **Mean difference (95% CI)** |
| --- | --- | --- | --- |
| **Women- Two months follow-up** | | | |
| Inpatient care between delivery and 30 days, hospital stay (days) mean (SE) | 0.064 (0.006) | 0.078 (0.007) | -0.014 (-0.027 to 0.018) |
| Inpatient care between 30 days and two months: hospital stay (days), mean (SE) | 0.071 (0.019) | 0.104 (0.026) | -0.033 (-0.096 to 0.030) |
| Outpatient care: number of visits, mean (SE) | 0.182 (0.012) | 0.197 (0.014) | -0.015 (-0.051 to 0.021) |
| Community health care: number of contacts, mean (SE) | | | |
| GP surgery visit | 1.068 (0.053) | 1.055 (0.040) | 0.013 (-0.117 to 0.143) |
| GP out-of-hours visit | 0.042 (0.007) | 0.038 (0.008) | 0.005 (-0.017 to 0.026) |
| Walk-in health centre visit | 0.048 (0.008) | 0.062 (0.017) | -0.014 (-0.051 to 0.020) |
| Practice nurse visit | 0.236 (0.049) | 0.159 (0.024) | 0.077 (-0.023 to 0.177) |
| Community nurse visit | 0.063 (0.015) | 0.025 (0.008) | 0.037*^1^ (0.004 to 0.071) |
| Physiotherapy visit | 0.071 (0.015) | 0.071(0.013) | <0.001 (-0.038 to 0.040) |
| NHS 111 call | 0.091 (0.010) | 0.099 (0.013) | -0.008 (-0.040 to 0.025) |
| Social worker visit | 0.021 (0.010) | 0.016 (0.008) | 0.005 (-0.021 to 0.031) |
| Other health professionals visit | 0.206 (0.035) | 0.220 (0.033) | -0.014 (-0.109 to 0.080) |
| The proportion of participants who received each class of drug (SE) | | | |
| Antibiotics | 0.087 (0.015) | 0.085 (0.013) | 0.002 (-0.009 to 0.023) |
| Pain killers | 0.107 (0.006) | 0.099 (0.008) | 0.008 (0.001 to 0.019) |
| Antidepressants | 0.061 (0.010) | 0.060 (0.012) | 0.001 (-0.007 to 0.016) |
| Other | 0.252 (0.039) | 0.249 (0.036) | 0.003 (-0.011 to 0.040) |
| **Women- Six months follow-up** | | | |
| Inpatient care between two and six months: hospital stay (days), mean (SE) | 0.020 (0.010) | 0.050 (0.016) | -0.030 (-0.068 to 0.007) |
| Outpatient care: number of visits, mean (SE) | 0.146 (0.012) | 0.160 (0.014) | -0.014 (-0.051 to 0.022) |
| Community health care: number of contacts, mean (SE) | | | |
| GP surgery visit | 0.799 (0.046) | 0.742 (0.045) | 0.057 (-0.070 to 0.184) |
| GP out-of-hours visit | 0.028 (0.008) | 0.029 (0.012) | <-0.001 (-0.029 to 0.028) |
| Walk-in health centre visit | 0.024 (0.007) | 0.026 (0.007) | 0.002 (-0.022 to 0.019) |
| Practice nurse visit | 0.179 (0.023) | 0.145 (0.018) | 0.034 (-0.023 to 0.091) |
| Community nurse visit | 0.021 (0.009) | 0.008 (0.004) | 0.013 (-0.008 to 0.033) |
| Physiotherapy visit | 0.111 (0.024) | 0.136 (0.031) | -0.025 (-0.103 to 0.052) |
| NHS 111 call | 0.061 (0.012) | 0.078 (0.017) | -0.017 (-0.058 to 0.025) |
| Social worker visit | 0.021 (0.010) | 0.005 (0.003) | 0.016 (-0.004 to 0.037) |
| Other health professionals visit | 0.215 (0.017) | 0.170 (0.040) | 0.045 (-0.061 to 0.152) |
| The proportion of participants who received each class of drug (SE) | | | |
| Antibiotics | 0.042 (0.007) | 0.051 (0.007) | -0.009 (-0.029 to 0.013) |
| Pain killers | 0.050 (0.006) | 0.065 (0.004) | -0.015 (-0.030 to 0.018) |
| Antidepressants | 0.059 (0.009) | 0.049 (0.007) | 0.010 (0.001 to 0.023) |
| Other | 0.131 (0.027) | 0.117 (0.021) | 0.014 (0.002 to 0.031) |
| **Infant- Two months follow-up** | | | |
| Inpatient care (readmission) within 30 days, hospital stay (days) mean (SE) | 0.148 (0.018) | 0.119 (0.015) | 0.029 (-0 .018 to 0.758) |
| Inpatient care between 30 days and two months: hospital stay (days), mean (SE) | 0.287 (0.069) | 0.348 (0.104) | -0.061 (-0.306 to 0.184) |
| Outpatient care: number of visits, mean (SE) | 0.445 (0.015) | 0.407 (0.016) | 0.038 (-0.006 to 0.083) |
| Community health care: number of contacts, mean (SE) | | | |
| GP surgery visit | 1.350 (0.046) | 1.360 (0.048) | -0.044 (-0.088 to 0.175) |
| GP out-of-hours visit | 0.096 (0.011) | 0.105 (0.013) | - 0.009 (-0.042 to 0.024) |
| Walk-in health centre visit | 0.101 (0.012) | 0.143 (0.017) | -0.042*^2^ (-0.082 to -0.001) |
| Practice nurse visit | 0.511 (0.028) | 0.532 (0.032) | -0.021 (-0.103 to 0.062) |
| Midwife visit | 2.572 (0.062) | 2.364 (0.065) | 0.208*^3^ (0.031 to 0.385) |
| Health visitor visit | 2.327 (0.048) | 2.410 (0.054) | -0.083 (-0.225 to 0.058) |
| Community nurse visit | 0.082 (0.016) | 0.071 (0.018) | 0.012 (-0.059 to 0.035) |
| Physiotherapy visit | 0.029 (0.007) | 0.018 (0.007) | 0.011 (-0.009 to 0.032) |
| Community paediatrician | 0.025 (0.005) | 0.027 (0.009) | -0.002 (-0.023 to 0.019) |
| NHS 111 call | 0.262 (0.018) | 0.294 (0.024) | -0.032 (-0.090 to 0.026) |
| Social worker visit | 0.042 (0.014) | 0.043 (0.013) | <-0.001 (-0.038 to 0.038) |
| Other health professionals visit | 0.169 (0.036) | 0.116 (0.020) | 0.052 (-0.029 to 0.134) |
| The proportion of participants who received each class of medication (SE) | | | |
| Antibiotic | 0.083 (0.009) | 0.069 (0.009) | 0.014 (0.011 to 0.034) |
| Other medications | 0.150 (0.011) | 0.125 (0.011) | 0.025 (-0.006 to 0.056) |
| **Infant- Six months follow-up** | | | |
| Inpatient care between two and six months: hospital stay (days), mean (SE) | 0.518 (0.242) | 0.275 (0.134) | 0.243 (-0.307 to 0.794) |
| Outpatient care: number of visits, mean (SE) | 0.294 (0.017) | 0.294 (0.018) | <-0.001 (-0.047 to 0.047) |
| Community health care: number of contacts, mean (SE) | | | |
| GP surgery visit | 1.104 (0.051) | 1.070 (0.060) | 0.033 (-0.122 to 0.189) |
| GP out-of-hours visit | 0.097 (0.012) | 0.111 (0.017) | -0.014 (-0.055 to 0.028) |
| Walk-in health centre visit | 0.121 (0.019) | 0.116 (0.018) | 0.005 (-0.045 to 0.055) |
| Practice nurse visit | 0.488 (0.035) | 0.561 (0.042) | -0.073 (-0.181 to 0.034) |
| Health visitor visit | 1.491 (0.047) | 1.408 (0.051) | 0.083 (-0.053 to 0.219) |
| Community nurse visit | 0.044 (0.012) | 0.042 (0.011) | 0.002 (-0.031 to 0.034) |
| Physiotherapy visit | 0.032 (0.012) | 0.021 (0.008) | 0.011 (-0.017 to 0.040) |
| Community paediatrician | 0.027 (0.009) | 0.019 (0.007) | 0.008 (-0.015 to 0.030) |
| NHS 111 call | 0.295 (0.022) | 0.303 (0.025) | -0.008 (-0.074 to 0.058) |
| Social worker visit | 0.019 (0.010) | 0.039 (0.026) | -0.019 (-0.074 to 0.034) |
| Other health professionals visit | 0.125 (0.029) | 0.080 (0.015) | 0.044 (-0.019 to 0.109) |
| The proportion of participants who received each class of medication (SE) | | | |
| Antibiotic | 0.086 (0.010) | 0.102 (0.012) | -0.016 (-0.048 to 0.016) |
| Other medications | 0.142 (0.012) | 0.145 (0.014) | -0.003 (-0.039 to 0.032) |

SE: Standard error

*^1^P-value= 0.038, *^2^ P-value= 0.038, *^3^ P-value= 0.022

***Table A4: NHS and PSS costs per protocol (Estimand 2) complete case- resource use data by treatment arm, and study period (2021–22 prices)***

| **Cost description** | **Between-arm differences cost (95% CI)**  (Induction group cost (N=341) – standard care group cost(N=228)) | **P-value** |
| --- | --- | --- |
| Woman’s antenatal | -35.13 (-51.67 to -18.60) | <0.00 |
| Delivery | 64.57 (-104.00 to 233.14) | 0.45 |
| Woman’s two months cost | -11.12 (-92.60 to 70.36) | 0.79 |
| Woman’s six months cost | 10.58 (-15.95 to 37.11) | 0.43 |
| Total Woman’s cost | 28.89 (-165.29 to 223.07) | 0.77 |
| Infant’s postpartum | 53.59 (-188.55 to 295.72) | 0.66 |
| Infant’s two months | 204.80 (-18.28 to 427.88) | 0.07 |
| Infant’s six months | 4.02 (-6080 to 68.84) | 0.90 |
| Total infant’s cost | 262.41 (-75.93 to 600.75) | 0.12 |
| Combined postpartum (delivery and infant’s postpartum) | 118.16 (-186.74 to 423.06) | 0.45 |
| Combined two months | 193.68 (-44.79 to 432.16) | 0.11 |
| Combined six months | 14.60 (-57.94 to 87.14) | 0.69 |
| Total combined cost (antenatal, combined postpartum, and two and six months follow up | 291.32 (-111.90 to 694.51) | 0.16 |

***Table A5: Cost-effectiveness: Incremental cost per case of prevented shoulder dystocia (2021-2022 prices), early induction compared with standard care- Subgroup analysis***

| **Scenario** | **Treatment arm, mean (SE) cost (£)** | | | | **Incremental cost (£)**  **(95% CI)** | **Treatment arm, mean (SE) prevented shoulder dystocia** | | **Incremental cases prevented shoulder dystocia (95% CI)** | **ICER (£)** | **Probability cost-effective at** | | | **NMBs** | | |
| --- | --- | --- | --- | --- | --- | --- | --- | --- | --- | --- | --- | --- | --- | --- | --- |
|  | N | **Early induction** | **N** | **Standard care** |  | **Early induction** | **Standard care** |  |  | **£5K** | **£10K** | **£20K** | **£5K** | **£10K** | **£20K** |

| **Subgroup Analysis^1^** |
| --- |

| Estimated Fetal weight centile >95% | 922 | 5,654.57 (104.29) | 923 | 5,535.10 (101.65) | 119.47  (-122.51 to 361.44) | 0.9748 (0.0055) | 0.9675 (0.0055) | 0.0073  (-0.0078 to 0.0223) | 16,399 | 0.29 | 0.39 | 0.55 | -83.00  (-373.00 to 206.00) | -47.00  (-364.00 to 271.00) | 26.00  (-386.00 to 438.00) |
| --- | --- | --- | --- | --- | --- | --- | --- | --- | --- | --- | --- | --- | --- | --- | --- |
| Estimated Fetal weight centile ≤95% | 525 | 5,200.81 (98.55) | 522 | 5,163.90 (102.15) | 36.90  (-199.52 to 273.33) | 0.9811 (0.0065) | 0.9731(0.0065) | 0.0080  (-0.0099 to 0.0258) | 4,618 | 0.51 | 0.60 | 0.70 | 3.00  (-291.00 to 297.00) | 43.00  (-289.00 to 375.00) | 123.00  (-333.00 to 579.00) |
| BMI <25 | 550 | 5,426.84 (115.88) | 550 | 5,215.45 (115.62) | 211.39 (52.96 to 475.74) | 0.9890 (0.0060) | 0.9710 (0.0060) | 0.0180 (0.0014 to 0.0346) | 11,750 | 0.14 | 0.30 | 0.63 | -191.00  (-534.00 to 152.00) | -101.00  (-475.00 to 274.00) | 80.00  (-396.00 to 556.00) |
| BMI ≥25 | 897 | 5,531.17 (100.77) | 895 | 5,512.48 (98.87) | 18.69  (-202.72 to 240.10) | 0.9696 (0.0058) | 0.9688 (0.0058) | 0.0008  (-0.0155 to 0.0171) | 24,032 | 0.46 | 0.47 | 0.49 | -15.00  (-296.00 to 266.00) | --11.00  (-322.00 to 300.00) | -3.00  (-417.00 to 411.00) |

^1^ For cost equation, there were no significant interaction terms between arm allocation and EFW centile (p-value= 0.38), and also between arm allocation and BMI (p-value= 0.77)

***Table A6: Cost-effectiveness: Incremental cost per maternal QALY gained (2021-2022 prices), Early induction compared with standard care- Subgroup analysis***

| **Scenario** | **Treatment arm, mean (SE) cost (£)** | | | | **Incremental cost (£) (95% CI)** | **Treatment arm, mean (SE) QALY** | | **Incremental QALYs**  **(95% CI)** | **ICER (£)** | **Probability cost-effective at** | | **NMBs** | |
| --- | --- | --- | --- | --- | --- | --- | --- | --- | --- | --- | --- | --- | --- |
|  | **N** | **Early induction** | **N** | **Standard care** |  | **Early induction** | **Standard care** |  |  | **£20K** | **£30K** | **£20K** | **£30K** |
| **Subgroup analysis^1^** | | | | | | | | | | | | | |
| Estimated fetal weight centile >95% | 922 | 5,654.57 (104.29) | 923 | 5,535.10 (101.65) | 119.47  (-122.51 to 361.44) | 0.4290 (0.0017) | 0.4290 (0.0020) | 0.0000  (-0.0037 to 0.0037) ^2^ | >30M^2^ | 0.23 | 0.25 | -119.00  (-434.00 to 195.00) | -119.00 (-462.00 to 224.00) |
| Estimated fetal weight centile <=95% | 525 | 5,200.81 (98.55) | 522 | 5,163.90 (102.15) | 36.90  (-199.52 to 273.33) | 0.4338 (0.0026) | 0.4277 (0.0030) | 0.0062  (-0.0014 to 0.0110) | 5,953 | 0.69 | 0.78 | 87.00 (-248.00 to 422.00) | 149.00 (-229.00 to 527.00) |
| BMI < 25 | 550 | 5,426.84 (115.88) | 550 | 5,215.45 (115.62) | 211.39 (52.96 to 475.74) | 0.4387 (0.0023) | 0.4257 (0.0025) | 0.0030 (-.0013 to 0.0073) | 70,277 | 0.21 | 0.27 | -151.00  (-514.00 to 212.00) | -121.00 (-517.00 to 275.00) |
| BMI>= 25 | 897 | 5,531.17 (100.77) | 895 | 5,512.48 (98.87) | 18.69  (-202.72 to 240.10) | 0.4259 (0.0019) | 0.4241 (0.0020) | 0.0018  (-0.0022 to 0.0057 | 10,584 | 0.54 | 0.58 | 17.00 (-289.00 to 322.00) | 34.00 (-300.00 to 368.00) |

^1^ For cost equation, there were no significant interaction terms between arm allocation and EFW centile (p-value= 0.38), and also between arm allocation and BMI (p-value= 0.77)

^2^ QALY difference = 0.0000038, ICER= £31,622,821

***Table A7 Labour induction vs Planned Caesarean section; NHS and PSS costs for complete case, study period, and cost category (2021–22 prices)***

| **Cost description** | **Adjusted^1^** | | | |
| --- | --- | --- | --- | --- |
|  | **Treatment group, mean (SE) cost (£)** | | **Between-group differences (95% CI)** | **p-value** |
|  | **Induction of labour (n=1,447)** | **Planned Caesarean section (n=274)** |  |  |
| Participants with complete data | N=403 | N= 76 |  | |
| **Woman’s cost** | | | | |
| **Antenatal phase** | | | | |
| Unscheduled hospital visit | 14.41 (2.87) | 43.50 (6.72) | -29.10 (-43.53 to 14.66) | <0.01 |
| Inpatient care | 5.04 (2.31) | -0.04 (5.41) | 5.08 (-6.54 to 16.70) | 0.39 |
| Medications | 0.50 (0.31) | 0.89 (0.72) | -0.39 (-1.94 to 1.16) | 0.62 |
| Total antenatal cost | 19.94 (3.72) | 44.35 | -24.41 (-43.12 to 5.70) | 0.01 |
| **Delivery cost** | 3,490.38 (46.34) | 3,442.26 (10.8.27) | 48.12 (-184.61 to 280.85) | 0.68 |
| **Postnatal cost- between delivery and two months follow-up** | | | | |
| Hospital readmission within 30 days of postnatal inpatient discharge | 57.49 (10.78) | -7.29 (31.93) | 64.79 (-3.84 to 133.42) | 0.06 |
| Inpatient care | 17.29 (12.20) | 76.05 (25.19) | -58.76 (-112.90 to 4.61) | 0.03 |
| Outpatient care | 36.81 (5.01) | 31.49 (11.72) | 5.32 (-19.87 to 30.50) | 0.68 |
| Community care | 60.91 (3.67) | 60.94 (8.57) | -0.03 (-18.45 to 18.39) | 0.10 |
| Medications | 5.97 (0.92) | 4.77 (2.16) | 1.20 (-3.44 to 5.84) | 0.61 |
| Total cost at two months follow-up | 178.48 (24.25) | 165.96 (56.67) | 12.52 (-109.29 to 134.33) | 0.84 |
| **Postnatal cost- between two- and six-months follow-up** | | | | |
| Inpatient care | 4.68 (5.71) | 33.64 (15.20) | -29.03 (-61.70 to 3.64) | 0.08 |
| Outpatient care | 31.58 (5.69) | 55.49 (13.29) | -23.91 (-52.49 to 4.66) | 0.10 |
| Community care | 47.70 (3.54) | 41.18 (8.28) | 6.52 (-11.27 to 24.33) | 0.47 |
| Medications | 5.20 (1.04) | 4.66 (2.44) | 0.54 (-4.70 to 5.78) | 0.84 |
| Total cost at six months follow-up | 89.10 (10.85) | 134.98 (25.36) | -45.88 (-100.38 to 8.62) | 0.10 |
| Woman’s total cost | 3,777.91 (54.07) | 3,787.56 (123.34) | -9.65 (-281.19 to 261.90) | 0.94 |
| **Baby’s cost** | | | | |
| **Baby’s postpartum** (additional care in the same hospital where they were born, transferred to a different hospital and additional care in a different hospital) | | | | |
| Inpatient critical care | 870.60 (75.00) | 836.11 (175.25) | 34.50 (-342.18 to 411.17)) | 0.86 |
| Transfer costs to a different hospital | 4.48 (3.79) | -1.95 (8.85) | 6.44 (-12.58 to 25.46) | 0.51 |
| Inpatient critical care in the different hospital | 10.44 (11.24) | 9.43 (26.26) | 1.01 (-55.44 to 57.45) | 0.97 |
| Total baby’s postpartum | 885.52 (76.37) | 843.58 (178.45) | 41.95 (-341.62 to 425.51) | 0.83 |
| **Baby’s two months follow-up** | | | | |
| Hospital readmission within 30 days of postnatal inpatient discharge | 129.54 (27.33) | 31.49 (76.89) | 97.80 (-67.47 to 263.08) | 0.25 |
| Inpatient care | 124.10 (41.42) | 44.99 (96.79) | 79.11 (-128.92 to 278.15) | 0.45 |
| Outpatient care | 131.74 (10.58) | 108.42 (24.72) | 23.32 (-29.81 to 76.45) | 0.39 |
| Community care | 214.27 (5.08) | 208.44 (11.88) | 5.83 (-19.71 to 31.37) | 0.65 |
| Medications | 5.28 (1.31) | 2.73 (3.06) | 2.55 (-4.04 to 9.13) | 0.44 |
| Antibiotic | 0.49 (0.15) | 0.81 (0.35) | -0.31 (-1.07 to -0.44) | 0.41 |
| Baby’s total cost at two months | 605.23 (72.56) | 405.07 (169.55) | 200.45 (163.98 to 564.89) | 0.28 |
| **Baby’s six months follow-up** | | | | |
| Inpatient care | 27.34 (13.55) | 43.05 (31.66) | -15.70 (-83.76 to 52.36) | 0.65 |
| Outpatient care | 79.37 (8.44) | 73.22 (19.72) | 6.14 (-36.24 to 48.54) | 0.78 |
| Community care | 100.98 (4.19) | 104.36 (9.80) | -3.37 (-24.44 to 17.69) | 0.75 |
| Medications | 10.16 (3.83) | 16.69 (8.94) | -6.53 (-25.75 to 12.69) | 0.51 |
| Antibiotic | 0.73 (0.24) | 1.20 (0.56) | -0.46 (-1.68 to 0.75) | 0.45 |
| Baby’s total cost at six months | 218.59 (19.28) | 238.52 (45.06) | -19.23 (-116.77 to 76.92) | 0.69 |
| Baby’s total cost | 1,709.65 (110.45) | 1,487 (258,07) | 222.48 (-332.22 to 777.17) | 0.43 |
| **Combined, woman and baby’s cost** | | | | |
| Intrapartum and postnatal period | 4,375.91 (91.54) | 4,285.84 (213.85) | 90.07 (-369.67 to 549.80) | 0.70 |
| Two months | 784.01 (77.14) | 571.04 (180.23) | 212.97 (-174.41 to 600.35) | 0.28 |
| Six months | 307.69 (24.01) | 373.49 (56.09) | -65.80 (-186.36 to -54.76) | 0.28 |
| Total cost | 5,487.56 (126.13) | 5,274.73 (294.71) | 212.83 (-420.62 to 846.28) | 0.51 |

^1^Adjusted with maternal age, fetal weight centile, and recruitment site.

***Table A8 Labour induction vs Planned Caesarean section; EQ-5D-5L score index; Complete case***

| **Quality of Life** | **Treatment group,**  **Mean (SE) EQ-5D index score** | | **Adjusted^1^** | |
| --- | --- | --- | --- | --- |
|  | **Induction of labour (n=1,447)** | **Planned Caesarean section (n=274)** | **Between-group differences (95% CI)** | **p-value** |
| Participants with complete data | N=403 | N= 76 |  | |
| Baseline | 0.7766 (0.0093) | 0.7763 (0.0217) | 0.0003 (-0.0464 to 0.0469) | 0.99 |
| Two months | 0.8861 (0.0062) | 0.8886 (0.0144) | -0.0025 (-0.0335 to 0.0285)) | 0.87 |
| Six months | 0.8818 (0.0072) | 0.8688 (0.0168) | 0.0130 (-0.0231 to 0.0490) | 0.48 |
| QALYs | 0.4332 (0.0028) | 0.4316 (0.0066) | 0.0016 (-0.0130 to 0.0160) | 0.83 |

^1^Adjusted for maternal age, fetal weight centile, and recruitment site.

***Table A9 Standard care vs Planned Caesarean section; NHS and PSS costs for complete case, study period, and cost category (2021–22 prices)***

| **Cost description** | **Adjusted^1^** | | | |
| --- | --- | --- | --- | --- |
|  | **Treatment group, mean (SE) cost (£)** | | **Between-group differences (95% CI)** | **p-value** |
|  | **Standard care (n=1,445)** | **Planned Caesarean section (n=274)** |  |  |
| Participants with complete data | N=302 | N= 76 |  | |
| **Woman’s cost** | | | | |
| **Antenatal phase** | | | | |
| Unscheduled hospital visit | 41.67 (4.88) | 46.24 (9.91) | 4.57 (-17.35 to 26.50) | 0.68 |
| Inpatient care | 16.27 (5.18) | 2.06 (10.52) | -14.21 (-37.48 to 9.07) | 0.23 |
| Medications | 0.87 (0.54) | 0.73 (1.11) | -0.14 (-2.60 to 2.32) | 0.91 |
| Total antenatal cost | 58.82 (7.50) | 49.4 (15.22) | -9.77 (-43.45 to 23.90) | 0.57 |
| **Delivery cost** | 3,435.63 (55.98) | 3,449.26 (113.63) | 13.63 (-237.84 to 265.10) | 0.92 |
| **Postnatal cost- between delivery and two months follow-up** | | | | |
| Hospital readmission within 30 days of postnatal inpatient discharge | 56.76 (12.30) | -0.76 (24.96) | -57.52 (-112.77 to -2.26) | 0.04 |
| Inpatient care | 38.96 (19.61) | 84.68 (39.80) | 45.71 (-42.37 to 133.79) | 0.31 |
| Outpatient care | 28.02 (4.66) | 33.47 (9.46) | 5.45 (-15.49 to 26.39) | 0.61 |
| Community care | 55.21 (3.77) | 62.60 (7.65) | 7.39 (-9.56 to 24.38) | 0.39 |
| Medications | 4.37 (0.63) | 4.94 (1.28) | 0.56 (-2.28 to 3.41) | 0.70 |
| Total cost at two months follow-up | 183.92 (27.79) | 184.97 (56.39) | 1.06 (-23.74 to 125.85) | 0.99 |
| **Postnatal cost- between two- and six-months follow-up** | | | | |
| Inpatient care | 16.50 (11.07) | 31.22 (22.47) | 14.73 (-35.01 to 64.47) | 0.56 |
| Outpatient care | 33.09 (7.28) | 57.43 (14.77) | 24.34 (-8.35 to 57.03) | 0.14 |
| Community care | 43.38 (3.64) | 41.78 (7.39) | -1.60 (-17.95 to 14.76) | 0.85 |
| Medications | 2.78 (0.61) | 4.43 (1.23) | 1.65 (-1.08 to 4.39) | 0.23 |
| Total cost at six months follow-up | 95.74 (15.31) | 134.86 (31.09) | 39.12 (-29.67 to 107.92) | 0.26 |
| Woman’s total cost | 3,774.10 (68.03) | 3,818.14 (138.10) | 44.04 (-261.58 to 349.66) | 0.78 |
| **Baby’s cost** | | | | |
| **Baby’s postpartum** (additional care in the same hospital where they were born, transferred to a different hospital and additional care in a different hospital) | | | | |
| Inpatient critical care | 713.57 (64.94) | 859.86 (131.82) | 146.28 (-145.45 to 438.19) | 0.33 |
| Transfer costs to a different hospital | 23.50 (9.82) | -6.17 (19.94) | -29.67 (-73.79 to 14.46) | 0.19 |
| Inpatient critical care in the different hospital | 23.49 (17.75) | -14.53 (36.04) | -38.02 (-117.78 to 41.73) | 0.35 |
| Total baby’s postpartum | 760.57 (67.64) | 839.16 (137.30) | 78.60 (-225.26 to 382.45) | 0.61 |
| **Baby’s two months follow-up** | | | | |
| Hospital readmission within 30 days of postnatal inpatient discharge | 33.36 (7.56) | 31.92 (15.35) | -1.44 (-35.41 to 32.53) | 0.93 |
| Inpatient care | 67.63 (21.03) | 50.36 (42.69) | -17.27 (-111.74 to 77.20) | 0.72 |
| Outpatient care | 86.10 (8.59) | 104.35 (17.44) | 18.29 (-20.32 to 56.89) | 0.35 |
| Community care | 210.17 (6.10) | 206.49 (12.32) | -3.68 (-30.94 to 23.59) | 0.79 |
| Medications | 3.30 (1.25) | 3.11 (2.54) | -0.19 (-5.80 to 5.42) | 0.95 |
| Antibiotic | 0.33 (0.12) | 0.88 (0.24) | -0.55 (0.02 to 1.08) | 0.04 |
| Baby’s total cost at two months | 400.79 (28.51) | 405.13 (57.87) | 4.33 (-123.73 to 132.39) | 0.95 |
| **Baby’s six months follow-up** | | | | |
| Inpatient care | 41.17 (18.73) | 50.19 (38.02) | 9.02 (-75.13 to 93.17) | 0.83 |
| Outpatient care | 66.53 (9.86) | 71.33 (20.01) | 4.80 (-39.48 to 49.08) | 0.83 |
| Community care | 104.56 (5.70) | 103.44 (11.58) | -1.11 (-26.74 to 24.51) | 0.93 |
| Medications | 5.74 (3.20) | 13.93 (6.50) | 8.19 (-6.18 to 22.57) | 0.26 |
| Antibiotic | 0.67 (0.25) | 1.41 (0.51) | 0.74 (-0.39 to 1.87) | 0.20 |
| Baby’s total cost at six months | 218.67 (24.63) | 240.31 (50.00) | 21.63 (-89.01 to 132.28) | 0.70 |
| Baby’s total cost | 1,380.03 (80.75) | 1,484.59 (163.92) | 104.58 (-258.21 to 467.33) | 0.57 |
| **Combined, woman and baby’s cost** | | | | |
| Intrapartum and postnatal period | 4,196.20 (90.74) | 4,288.42 (184.20) | 92.22 (-315.42 to 499.87) | 0.66 |
| Two months | 584.71 (41.25) | 590.09 (83.75) | 5.38 (-179.95 to 190.72) | 0.95 |
| Six months | 314.41 (29.54) | 375.17 (59.96) | 60.76 (-71.93 to 193.45) | 0.37 |
| Total cost | 5,154.13 (109.85) | 5,302.72 (223.00) | 148.59 (-344.92 to 642.11) | 0.55 |

^1^Adjusted with maternal age, fetal weight centile, and recruitment site.

***Table A10 Standard care vs Planned Caesarean section; EQ-5D-5L score index; Complete case***

| **Quality of Life** | **Treatment group,**  **Mean (SE) EQ-5D index score** | | **Adjusted^1^** | |
| --- | --- | --- | --- | --- |
|  | **Standard care (n=1,445)** | **Requesting planned Caesarean section (n=274)** | **Between-group differences (95% CI)** | **p-value** |
| Participants with complete data | N=302 | N= 76 |  | |
| Baseline | 0.7957 (0.0106) | 0.7744 (0.2149) | -0.0214 (-0.0689 to 0.0262) | 0.38 |
| Two months | 0.8867 (0.0082) | 0.8753 (0.0168) | -0.0114 (-0.0486 to 0.0258) | 0.55 |
| Six months | 0.8823 (0.0087) | 0.8572 (0.0176) | -0.0251 (-0.0641 to 0.0138) | 0.21 |
| QALYs | 0.4351 (0.0063) | 0.4262 (0.0074) | -0.0088 (-0.0251 to 0.0075) | 0.29 |

^1^Adjusted for maternal age, fetal weight centile, and recruitment site.
